# From Understanding Mechanical Behavior to Curvature Prediction of Humidity‐Triggered Bilayer Actuators

**DOI:** 10.1002/adma.202007982

**Published:** 2021-01-20

**Authors:** Carsten Dingler, Henry Müller, Matthias Wieland, Dominik Fauser, Holger Steeb, Sabine Ludwigs

**Affiliations:** ^1^ IPOC–Functional Polymers Institute of Polymer Chemistry University of Stuttgart Pfaffenwaldring 55 70569 Stuttgart Germany; ^2^ Institute of Applied Mechanics (Civil Engineering) & SC SimTech University of Stuttgart Pfaffenwaldring 7 70569 Stuttgart Germany

**Keywords:** bilayer actuators, curvature prediction, humidity trigger, mechanical properties, PEDOT:PSS

## Abstract

Nature will always be an endless source of bioinspiration for man‐made smart materials and multifunctional devices. Impressively, even cutoff leaves from resurrection plants can autonomously and reproducibly change their shape upon humidity changes, which goes along with total recovery of their mechanical properties after being completely dried. In this work, simple bilayers are presented as autonomously moving, humidity‐triggered bending actuators. The bilayers—showing reproducible bending behavior with reversible kinematics and multiway behavior—are studied in terms of their mechanical behavior upon humidity changes. The active layer consists of a highly conducting polymer film based on poly(3,4‐ethylenedioxythiophene):poly(styrene sulfonate) (PEDOT:PSS) with poly(dimethylsiloxane) (PDMS) as passive layer. The response to humidity is explored with dynamic mechanical thermal analysis and quartz crystal microbalance measurements. Introduction of a composite beam model allows to predict the curvature of the actuators with input from the rheological measurements. It is clearly demonstrated that volumetric strain and Young's modulus, both heavily influenced by the water uptake, dominate the bending behavior and therefore the curvature of the actuators. This loop of rheological characterization coupled with an analytical model allows to predict curvatures of in principle any complex geometry and material combination for moving parts in soft robotics.

## Graphical abstract

An in‐depth mechanistic understanding of the mechanical properties of bilayer actuators based on the humidity‐responsive polymer poly(3,4‐ethylenedioxythiophene):poly(styrene sulfonate) (PEDOT:PSS) in combination with volumetric strain measurements allows for the curvature prediction of humidity‐triggered bilayer actuators by an analytic composite beam model.

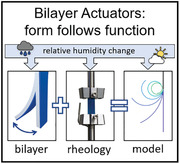

## 1 Introduction

Conjugated polymers have attracted great interest for the use in flexible electronic devices as they combine the advantages of commodity polymers with a variety of functional optical and electronic properties.^[^ ^1^ ^]^ The responsiveness to stimuli such as heat, electric voltage, pH, light, and humidity have further sparked fascination for their use as smart materials, which implies electronic skin (e‐skin) and soft robotics applications such as sensors and actuating devices.^[^ ^2^ ^]^

**Figure 1:**
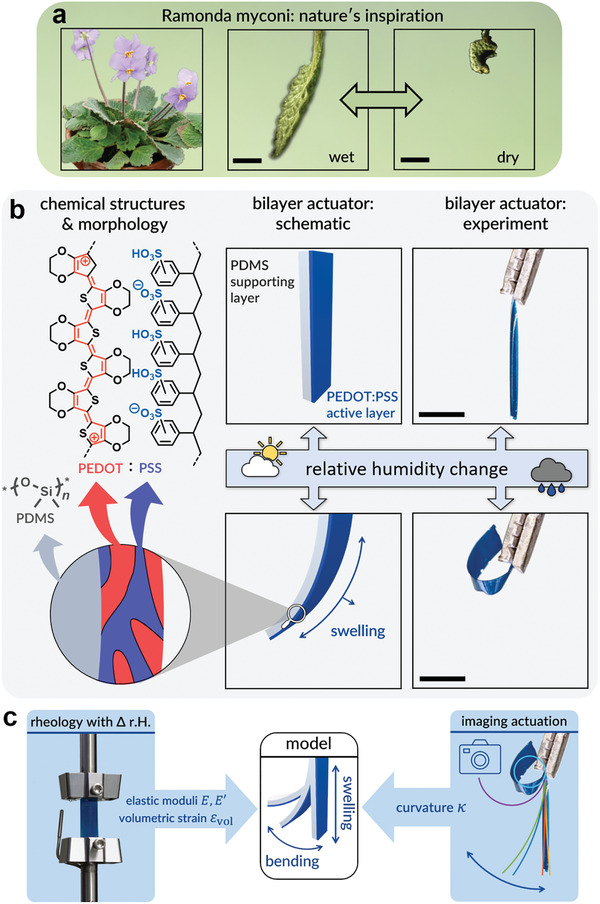
Concept and materials. a) Photograph of the desiccation tolerant plant *Ramonda myconi* and adaptation of a cutoff leave during drying (scale bar 1 cm). Adapted under the terms of the CC‐BY 4.0 license.^[^ ^4^ ^]^ Copyright 2018, The Authors, published by Frontiers. b) Left: Chemical structures and simplified depiction of the phase‐separated morphology of PEDOT:PSS. Center: Schematic of a PEDOT:PSS/PDMS bilayer in the dry state and illustration of the bending after swelling at high r.H. Right: Actuator experiment with a 17 µm thick bilayer in the dry (r.H. = 0.01%) and wet (r.H. = 88%) state (scale bar 2 mm). c) Scheme of the concept of this work. Actuation was analyzed by imaging to extract curvatures κ. Rheological measurements were conducted to determine mechanical properties of the layers. Those could be used in a model to predict actuation, which was compared to the experimental results.

Mechanical response to changing humidity is a phenomenon well‐known from nature, e.g., from the scales of a pine cone,^[^ ^3^ ^]^ and resurrection plants such as *Ramonda myconi* (**Figure** **1**a).^[^ ^4^ ^]^ Even cutoff leaves bend and contract in a complex manner upon desiccation (Figure 1a), and can be fully recovered in terms of shape and mechanical properties upon water contact.^[^ ^4^ ^]^ The plants share the same origin of the bending movements, namely structural features that behave as effective bilayers with one layer expanding more strongly than the other one upon contact with water.^[^ ^3^, ^4^ ^]^

From bioinspiration to conducting‐polymer devices, first actuators were based on electropolymerized polypyrrole films, driven by electrochemical triggers^[^ ^5^ ^]^ as well as Joule heating and humidity changes.^[^ ^6^ ^]^ The inherent multifunctionality and water processability has made the use of poly(3,4‐ethylenedioxythiophene):poly(styrene sulfonate) (PEDOT:PSS) as active layer material intriguing. The morphology of PEDOT:PSS was comprehensibly discussed and theoretically investigated by Modarresi et al.^[^ ^7^ ^]^ and is known to depend strongly on processing conditions.^[^ ^8^ ^]^ The dissimilar nature of the two components, doped PEDOT and the polyelectrolyte PSS, causes a co‐continuous morphology (cf. Figure 1b) with PEDOT rich and PSS rich domains and a granular structure in the 10–40 nm range.^[^ ^8^, ^9^ ^]^ Apart from devices that require the high electronic conductivity of PEDOT:PSS,^[^ ^10^ ^]^ a number of iontronic applications have been demonstrated.^[^ ^11^ ^]^ The latter are based on independent pathways allowing for both electronic and ionic charge transport, which also showed to depend on the humidity of the environment.^[^ ^12^ ^]^ In addition, it was demonstrated that actuating devices based on PEDOT:PSS with and without poly(dimethylsiloxane) (PDMS) as substrate could be actuated by Joule heating and humidity.^[^ ^13^, ^14^ ^]^

It is reasonable to correlate the movement of such actuators to their mechanical behavior. The mechanical behavior, however, also depends on the environmental conditions, more specifically on the relative humidity (r.H.). Lang et al.^[^ ^15^ ^]^ indicated this for PEDOT:PSS, but only measured at around ambient conditions (r.H. range from 23% to 50%). In literature, elastic moduli such as Young's modulus of PEDOT:PSS vary strongly and range over at least one order of magnitude from 0.28 to 3.6 GPa.^[^ ^15^, ^16^ ^]^ It is not surprising that the absolute values differ so strongly since they depend on numerous parameters. Among them are the precise sample composition, i.e., batch type and additives, processing conditions, but also the sample geometry, and the measurement details such as method and—not to be neglected—the environmental conditions. The humidity in the environment has, for example, also shown to be a key parameter for optoelectronic,^[^ ^17^ ^]^ thermoelectric,^[^ ^18^ ^]^ and piezoresistive^[^ ^19^ ^]^ properties of PEDOT:PSS.

In this work, a simple bilayer architecture with a single layer of PEDOT:PSS on top of a PDMS passive layer is used, and the humidity‐triggered actuation is studied as well as the humidity dependent rheological properties of these bilayers in the very same geometry. The Young's modulus of the active PEDOT:PSS layer is shown to depend strongly on humidity, particularly in the very high (r.H. > 80%) and very low (r.H.  ≤ 10%) range. This trend is also perfectly reflected in the course of the water uptake and the volumetric strain as a function of r.H. Feeding the obtained humidity‐dependent rheological characteristics together with the geometrical data into a very simple analytical model based on Timoshenko's equation for composite beams^[^ ^20^ ^]^ allows to predict curvatures of the bilayer actuators and vice versa, the feedback loop of which is given in Figure 1c.

Expanding this approach will open the access to predictions of more complex geometries, can guide the choice of material combinations for smart devices for soft robotics and can be extended to other triggers such as temperature or electric fields.

## 2 Results and Discussion

**Figure 2:**
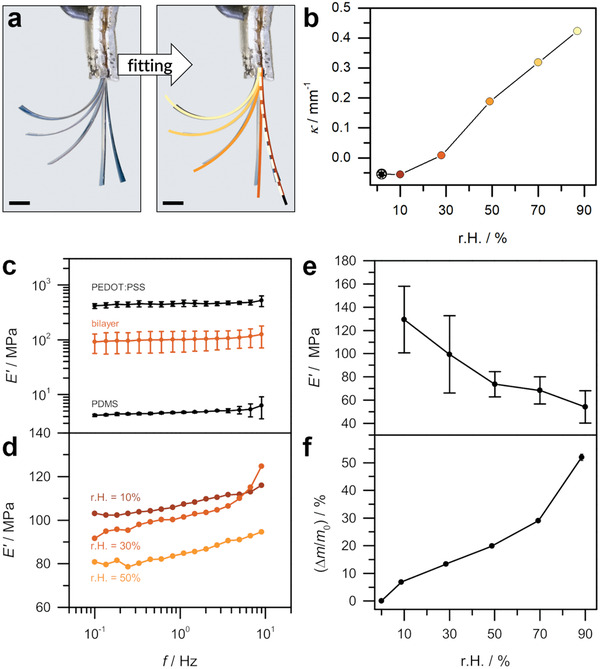
Actuation and mechanical properties. a) Actuator movement of a PEDOT:PSS/PDMS bilayer (54 µm thickness) in response to changing relative humidity (scale bar 1 mm). Segments of circles were fitted to the images to extract the curvature κ as a function of r.H. as shown in (b). Colors in (a) correspond to those in (b). c) Averaged frequency sweeps of bilayers (17 µm thickness), PEDOT:PSS single layers, and PDMS (thickness 65 µm, with plasma and 140 °C treatment) films (all at r.H. = 30%). d) Averaged frequency sweeps of bilayers (17 µm thickness) at selected relative humidities. Error bars are omitted for clarity. e) Humidity dependence of the averaged storage modulus *E'* of 17 µm bilayers as extracted from humidity sweep experiments. f) Averaged water uptake Δ*m*/*m*_0_ of PEDOT:PSS as a function of relative humidity, determined by QCM. All measurements were performed at 30 °C and all error bars represent standard deviations calculated from multiple measurements. All rheological measurements were conducted with the UXF system.

**Figure** **2**a shows the actuation of a 54 µm thick bilayer comprising a ≈6 µm PEDOT:PSS and a 48 µm PDMS layer. The PEDOT:PSS was prepared following a protocol which involves the addition of ethylene glycol to enhance the electronic conductivity.^[^ ^8^ ^]^ The active layer film thickness is selected to be large enough to be able to efficiently measure rheological properties but also thin enough to enable fast diffusion of water into the film to achieve homogeneous swelling and short response times. The thicknesses were measured by profilometry at ambient humidity.

The relative humidity in the actuator testing experiment was increased stepwise from 2% to 87% and then set back to 2% (cf. setup in Figure S1 in the Supporting Information). The actuator is slightly bent to the right in the initial state at r.H. = 2% and then considerably bends to the left as the humidity is increased. Curvatures κ were extracted from the images by fitting a circle to the bent actuators and calculated as the reciprocals of the radii, κ = 1/*R* (Figure 2a, cf. also Figure S2 in the Supporting Information). The compilation of fits indicates perfectly reversible kinematics, without any observable hysteresis, as initial and final state can barely be distinguished. The gradual change in shape of these bilayer actuators in response to a stepwise variation in humidity underlines the potential of this material system for the use in, e.g., humidity sensors. The course of the curvature during the whole measurement series nicely follows the gradual increase in relative humidity (Figure S3, Supporting Information). The trend of the curvature as a function of relative humidity in Figure 2b shows that the curvature increases up to r.H. = 30%, surges when the humidity was set to 50%, and further increases up to a maximum of κ = 0.42 mm^–1^ at r.H. = 90%.

Thinner bilayers with a total thickness of 17 µm (with identical 6 µm thick PEDOT:PSS, but 11 µm thick PDMS) tended to bend more strongly with a steep increase in curvature between 50% and 90% r.H. (Figures S4 and S5, Supporting Information). A maximum curvature of 1.2 mm^–1^ was reached, which is about three times the value of the 54 µm bilayer at r.H. = 90%. All bilayers showed a multiple‐way effect, i.e., they could be cycled reversibly and repeatedly (cf. Figure S6 in the Supporting Information). When samples were removed from the cell at high humidity (i.e., an abrupt change from r.H. = 90% down to ambient conditions, which is often around 30% r.H.), they instantly started bending backward. This illustrates that these actuators do autonomously adapt to the surrounding conditions within seconds. However, as the environmental chamber is slow to reach a certain preset r.H., actuation speed or frequency in response to humidity could not be quantified.

The bending of the actuators is the consequence of swelling of the PEDOT:PSS layer by taking up water via a diffusion process from the environment (cf. Figure 1b). As the PDMS film remains passive, the expansion of the active layer produces stresses in the bilayer, which are minimized by the bending movement. To quantify the extent of swelling, the water uptake of thin PEDOT:PSS films was determined by quartz crystal microbalance (QCM) measurements (cf. Figure S1 in the Supporting Information). After initial drying, the humidity was increased stepwise up to r.H. = 90% and the frequency change of the oscillating polymer film‐covered quartz crystal measured. According to the Sauerbrey equation,^[^ ^21^ ^]^ a decrease in frequency can be related to an increase in mass, from which the relative water uptake can be calculated. The resulting relative mass increase Δ*m*/*m*_0_ as a function of relative humidity r.H. is given in Figure 2f and Table S1 (Supporting Information). The course of the curvature resembles a type II sorption isotherm^[^ ^22^ ^]^ similar to those also observed in literature of other sulfonate‐based polyelectrolyte materials.^[^ ^23^ ^]^ This implies three stages of water sorption: The first short and steep increase in mass from 0 to (6.9 ± 0.2) wt% at r.H. = 10% can be attributed to the formation of a water monolayer on the internal surface of PEDOT:PSS. This is followed by a linear regime roughly up to r.H. = 50% giving a water uptake of (19.9 ± 0.7) wt%, which can be related to multilayer formation. Above r.H. = 70%, a steep rise up to a maximum of (51.9 ± 1.2) wt% at r.H. = 90% is found, which is a consequence of the sorption as liquid water. It can be estimated that this corresponds to ≈1 water molecule per free sulfonate group at r.H. = 10%, ≈2 to 4 in the linear range, and 9 at r.H. = 90% (cf. Supporting Information). While the general trend of the water sorption curve matches with that of our^[^ ^12^ ^]^ and other groups,^[^ ^24^ ^]^ absolute values can differ as they depend on batch, film preparation (including use of additives), and the measurement technique. Experiments by us (not shown) and others^[^ ^25^ ^]^ indicate that the PDMS layer can be regarded as passive and its properties independent of humidity.

The mechanical behavior of the bilayers and of PEDOT:PSS single layers is strongly dependent on r.H. as demonstrated by dynamic mechanical thermal analysis (DMTA) using an environmental chamber to control temperature and humidity (cf. Figure S1, Supporting Information). This is a powerful method to characterize the effective linear viscoelastic properties of polymer materials exposed to substantially varying environmental conditions using small amplitudes.

Transferring rheological characterization methods to rather thin samples is technically not trivial. Small samples tend to show a size and shape dependent response in experiments. Applying tensile tests with carefully selected sample geometries, force (or stress) amplitudes could be adopted to the measurement accuracy of the chosen DMTA device.

Typically, amplitude sweeps were used to determine the linear viscoelastic range of the samples. Subsequent frequency sweeps were measured to analyze frequency (or time) dependent viscoelastic behavior in the form of the complex modulus E* = *E*’ + i*E*''. As the loss modulus *E''* (viscous part) was at least one order of magnitude lower than the storage modulus *E'* (elastic part) in all experiments (cf. Figure S9 in the Supporting Information) and both components were rather independent of frequency in the investigated range, the materials were regarded as mainly elastic. This implies that *E'* ≈ |E*| ≈: *E*. Young's modulus is therefore discussed as a measure of the effective mechanical stiffness of the materials.

The averaged frequency sweep of 17 µm thick bilayers at r.H. = 30% is displayed in Figure 2c. The storage modulus *E'* is roughly 110 MPa and rather constant in the range from 0.1 to 10 Hz. For comparison, the average frequency sweep of PEDOT:PSS single layers at r.H. = 30% gives values around 400 to 500 MPa (Figure 2c). As mentioned above, the absolute elastic moduli of PEDOT:PSS strongly differ in literature. The PEDOT:PSS used in this study has a PEDOT‐to‐PSS ratio of 1:2.5 (w/w) and was prepared with ethylene glycol (EG) as additive in order to increase the conductivity as commonly reported in literature.^[^ ^8^ ^]^ It mainly enhances phase separation and increases the size of PEDOT rich domains,^[^ ^8^ ^]^ which results in a conductivity of PEDOT:PSS of around 500 S cm^–1^ at ambient r.H. in the bilayers of this work (four‐point probe measurements, data not shown). This was supposed to keep options open for the use as electrochemomechanical actuators, actuators controlled by Joule heating, or actuators in other electronically connected devices in future studies. It should be noted that the electronic conductivity of PEDOT:PSS (with a weight ratio of 1:2.5) was shown to be rather independent of r.H.^[^ ^12^, ^26^ ^]^ Comparison of these DMTA measurements with PEDOT:PSS films prepared without EG clearly show that the inclusion of EG also has a softening effect (Figure S15, Supporting Information), probably as EG remaining in the film can compete with polar intermolecular interactions in PEDOT:PSS.

The rheological characterization of pure PDMS samples was technically challenging by the fact that 11 µm PDMS films were too thin to be handled in DMTA experiments as they immediately wrinkled and stuck together when standing freely and ruptured at attempts to unwrinkle. Instead, PDMS samples with thicknesses ranging from 65 to 220 µm were tested at r.H. = 30%. The average frequency sweep of a 65 µm thick film is included in Figure 2c. It gives a storage modulus of 4–5 MPa, which is in the typical literature range.^[^ ^27^ ^]^ The thickness dependent measurements suggest that the storage modulus increases with decreasing PDMS thickness (Figure S10, Supporting Information). It can be inferred that 48 µm thick PDMS should have a storage modulus of around 5 MPa and 11 µm thick PDMS around 10 MPa (cf. discussion in the Supporting Information). Variation of the PDMS thickness between 11 and 48 µm in bilayers with a constant PEDOT:PSS thickness also indicates a decreasing storage modulus with increasing bilayer thickness (Figure S13, Supporting Information).

Taken together, the measurements of single PEDOT:PSS and PDMS layers in comparison with the bilayers clearly show that the rheological behavior of the bilayers is based on a combination of the properties of the individual layers. The domination of the storage moduli over the loss moduli and the rather frequency independent courses indicate that Young's modulus is a quantity describing the mechanical behavior of PEDOT:PSS and PDMS sufficiently well in a broad frequency range.

Focusing on humidity dependence, the frequency sweeps of 17 µm bilayers at r.H. = 10%, 30%, and 50% are shown in Figure 2d. The *E'* curve clearly shifts downward when r.H. is increased. This implies that the storage modulus at a given frequency decreases with increasing humidity. As the storage moduli appeared rather independent of frequency for r.H. = 10%–50%, humidity sweeps were performed at 1 Hz in the full r.H. range from 10% to 90%. In the measurements the relative humidity was increased stepwise with a waiting time of 0.5–2 h for equilibration of the samples between the individual steps. The average of the measurements of multiple samples is given in Figure 2e. It can be seen that *E'* decreases from about 130 to 74 MPa when going from r.H. = 10% to 50%. When further raising r.H. to 90%, the storage modulus eventually drops to 54 MPa. This leads to a decrease in *E'* by a factor of 2.4 from lowest to highest humidity. The decrease in storage modulus with increasing relative humidity is also perfectly in line with the QCM measurements, indicating continued water uptake until high humidities. Regarding stiffness, it can be followed that the PEDOT:PSS/PDMS bilayer material tends to get softer as humidity increases. It can be deduced that the uptake of water has a softening effect on the bilayers, which can clearly be ascribed to the behavior of the PEDOT:PSS active layer.

**Figure 3:**
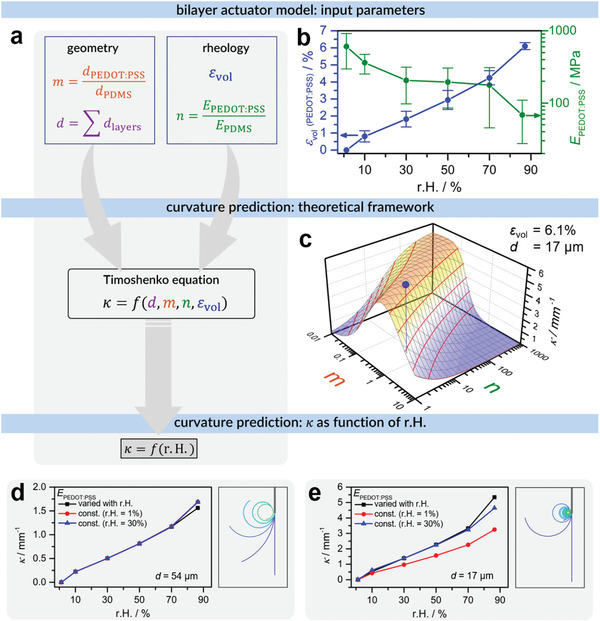
Actuator model. a) Schematic description of the actuator model with model input parameters, Timoshenko's composite beam equation as a theoretical framework to calculate the curvature κ, and the prediction of the actuator bending (κ) based on the humidity dependence of the mechanical properties. b) Volumetric strain ε_vol_ and Young's modulus *E* of PEDOT:PSS as a function of r.H. as determined by rheology (SRF system). c) 3D plot of the curvature κ as a function of the thickness ratio *m*  =  *d*_PEDOT:PSS_/*d*_PDMS_ and the ratio of Young's moduli *n*  =  *E*_PEDOT:PSS_/*E*_PDMS_ according to the Timoshenko^[^ ^20^ ^]^ Equation (1). A total thickness of *d*  =  17 µm and a volumetric strain of ε_vol_  =  6.1% were used for the calculation. Additionally, the data point at the experimental values *m* = 0.48 and *n* = 6.8 is highlighted as a blue dot, with a projection to add clarity. d) Prediction of κ(r.H.) for a 54 µm bilayer according to the model and visualization of a hypothetical actuator of length 5.0 mm. e) Corresponding prediction of a 17 µm bilayer (length 5.5 mm). The thickness of the hypothetical actuators is not true to scale as the actuators would not be visible otherwise. Error bars in (b) represent standard deviations of the average of measurements with three different samples.

Based on this complete material characterization of the bilayers and its components, a direct correlation between mechanical properties and the bending movements of the bilayer is established in the following. A composite beam model is used to predict the curvature of an actuator from input parameters from both geometry and rheology. The model is analytical and based on Timoshenko's equation of a bilayer beam

(1) $$\begin{array}{c} \kappa \;=\frac{\varepsilon_{\text{vol}}}{d}\;\;\cdot \frac{6{\left(1+m\right)}^{2}}{3{\left(1+m\right)}^{2}+\left(1+mn\right)\cdot \left(m^{2}+\frac{1}{mn}\right)} \end{array}$$

initially developed within the framework of linear beam theory with volumetric strains caused by thermal expansion.^[^ ^20^ ^]^ The equation can, however, be used more generally for bending caused by any other origins of volumetric strain as well.^[^ ^14^, ^28^ ^]^ According to this equation, the curvature κ could be calculated from the volumetric strain ε_vol_, the thickness ratio *m* = *d*_PEDOT:PSS_/*d*_PDMS_ of the two layers, the ratio of Young's moduli *n* = *E*_PEDOT:PSS_/*E*_PDMS_, and the total thickness *d* = *d*_PEDOT:PSS_ + *d*_PDMS_ (**Figure** **3**a).

The remaining volumetric strain ε_vol_ of PEDOT:PSS was determined in a second set of rheological measurements. This quantity corresponds to the linear extension of the samples caused by swelling with water at increasing r.H. Loading the samples with force ramps at distinct humidities enabled the measurement of strains as response. From a mechanical point of view, volumetric strains are the governing kinematical quantities of the water uptake process. During water uptake, the shape of the material is preserved while the volume of the material is evolving. Thus, assuming small strains (linear kinematics), the strains can be additively decomposed into a deviatoric part and a volumetric part, the latter denoted as ε_vol_. The deviatoric part can be neglected for the water uptake mechanism. In the present experimental investigations, volumetric strains were carefully measured relative to the dry state in its initial configuration (initial length *l*_0_) at r.H. = 1%. In addition, these measurements also provided Young's modulus *E*_PEDOT:PSS_ as the ratio of stress and strain (Figures S16 and S17, Supporting Information).

The resulting quantities ε_vol_ and *E*_PEDOT:PSS_ as a function of r.H. are plotted in Figure 3b. A short steep rise in volumetric strain can be seen from 0 to 10% r.H., adopting a value of ε_vol_ = 0.8%. The volumetric strain further increases rather linearly with values of 1.8%, 2.9% and 4.2% at r.H. = 30%, 50%, and 70%, respectively. Finally, it surges to a maximum of 6.1% at r.H. = 90%.

Comparing to literature, Okuzaki et al. report a maximum volumetric strain for PEDOT:PSS of 4.5%, assuming the initial length of the samples at r.H. = 20%.^[^ ^13^ ^]^ However, the maximum volumetric strain was probably underestimated since there are also strong changes in the low‐humidity range as shown in Figure 2f. In the work of Taccola et al.^[^ ^14^ ^]^ the authors measured curvatures of actuator movements based on Joule heating and calculated volumetric strains of around 2% using Timoshenko's equation. In our work, however, ε_vol_ was directly measured and fed into a model to allow predictions on the curvatures. The volumetric strain is a material characteristic which results from volume changes due to humidity variations. In the small strain regime the volumetric strain caused by humidity changes is superimposed on the mechanical strains. Thus, rheological measurements and complementary volumetric strain measurements allow to predict kinematics of actuators.

Interestingly, the overall course of the volumetric strain over the whole relative humidity range in Figure 3b perfectly resembles the water sorption curve determined by QCM. This seems also reasonable as both describe the uptake of water, yet as a change in mass on the one hand (QCM) and a change in volume (restricted to one dimension) on the other hand (volumetric strain).

Young's modulus is 605 MPa at r.H. = 1%, decreases to about 200 MPa at r.H. = 30%–70% and drops to 68 MPa at the highest r.H. This is almost an order of magnitude lower compared to the dry state, yet mechanical properties are commonly only measured at ambient r.H., which means ≈30%, or in a dry gas atmosphere which corresponds to r.H. ≈ 1%. The evolution of Young's modulus *E* of neat PEDOT:PSS seems to be fairly comparable to that of the storage modulus *E'* of the bilayers (Figure 2e). Both moduli exhibit a strong decrease in the lower humidity range, remain rather constant in the mid‐range, and then plunge when the humidity is increased to about 90%. This implies that the strongest changes could be observed in the low and high‐humidity range.

Knowing the humidity dependence of ε_vol_ and the rheological properties expressed by *n* as well as the sample geometry defined by *m* and *d*, the curvature of the bilayer sample can be predicted according to Equation (1). Generally speaking, the predicted curvature most strongly depends on the volumetric strain ε_vol_ and the sample thickness *d* (Figure S18a,b, Supporting Information) as κ ∝ ε_vol_ and κ ∝1/*d* (cf. Equation 1). In Figure 3c the general dependence of the curvature κ on the other two influencing parameters, namely thickness ratio *m* and moduli ratio *n*, is plotted in a 3D graph. The thickness was set to *d* = 17 µm, corresponding to the thinner bilayer in Figure 1 and of the rheological measurements in Figure 2. The volumetric strain was fixed at ε_vol_ = 6.1% to give the maximum curvature as at r.H. = 90%.

For *m* < 1, the thickness of the active layer is smaller than that of the passive layer. This is beneficial as thin active layers enable short response times by fast diffusion processes throughout the layer and thick passive layers provide solid support. In this range, the curvature increases with increasing moduli ratio *n* (as long as *m* and *n* are not too large, cf. contour plot in Figure S21, Supporting Information). This implies that high curvatures can mainly be reached when the active layer is stiffer than the passive layer. For thicker bilayers (*d* = 54 µm), the dependence on *m* and *n* is the same, (Figure S19, Supporting Information) yet overall curvatures are lower because of the strong dependence of κ on *d* (cf. Figure S18b, Supporting Information).

The maximum curvatures for the specific bilayers investigated in this work can be predicted by using the values ε_vol_ = 6.1% and *E*_PEDOT:PSS_ = 68 MPa measured at r.H. = 90%. For a 17 µm bilayer with thickness ratio *m* = 0.48 (*d*_PEDOT:PSS_/*d*_PDMS_ ≈ 6 µm/11 µm) and moduli ratio *n* = 6.8 (*E*_PEDOT:PSS_/*E*_PDMS_ = 68 MPa/10 MPa), the maximum curvature would be 5.3 mm^–1^. For a 54 µm bilayer actuator, a maximum curvature of 1.6 mm^–1^ could be calculated with *m* = 0.11 (*d*_PEDOT:PSS_/*d*_PDMS_ ≈ 6 µm/48 µm) and *n* = 13.6 (*E*_PEDOT:PSS_/*E*_PDMS_ = 68 MPa/5 MPa). The global maxima generally achievable for 17 and 54 µm bilayers would be κ = 1.7 and 5.4 mm^–1^, respectively. To obtain maximum curvatures, the selected experimental bilayer geometries therefore appear to be close to the optimum, regarding choice of *m* and *n* (cf. Figures S20 and S22, Supporting Information).

Including the humidity dependence of ε_vol_ and *E*_PEDOT:PSS_ (from Figure 3b) in the model (Equation 1) yields the curvature of both the 54 and 17 µm actuators as a function of r.H. as shown in Figure 3d,e. It is clearly visible that the curvature increases with increasing relative humidity. The courses closely resemble the trend of the volumetric strain in Figure 3b, which is the factor with greatest influence as mentioned before.

The humidity dependence of *E*_PEDOT:PSS_ showed to be a crucial parameter especially for the 17 µm bilayers. The maximum curvature predicted from a humidity dependent *E*_PDOT:PSS_ is 5.3 mm^–1^ (cf. Figure 3e). If a constant *E*_PEDOT:PSS_ with the value taken at r.H. = 30% is used, i.e., as if rheology was measured only at ambient r.H., the curvature at r.H. = 90% would be 4.6 mm^–1^. If the value was taken at r.H. = 1%, which corresponds to a dry gas atmosphere, the curvature would be even lower with a value of 3.2 mm^–1^. This clearly underlines that it is highly important to measure the mechanical properties of the materials as a function of humidity to be able to predict actuator behavior more reliably.

The actuator curvatures in the graphs in Figure 3d,e are also visualized using the same lengths as the experimental actuators in Figure 2 and Figure S4 (Supporting Information). With increasing r.H., which means increasing ε_vol_ and decreasing *E*_PEDOT:PSS_, the simulated actuators start bending gradually, just as in the experimental case. The 54 µm actuator reaches a final state at the highest r.H. where it forms more than a full cycle with about 1.2 windings. The 17 µm actuator even winds up almost fourfold as much with a number of 4.7 windings. Overall, the analytically calculated curvatures nicely predict the trends of the experimental actuators. It has to be noted, however, that there is an inherent linear assumption in the Timoshenko equation. Even if observed maximum volumetric strains are small (max(ε_vol_) ≈ 6%), the observed rotations and therefore the curvatures become large, particularly at maximum relative humidities. Applying more sophisticated beam models which take into account moderately large or even large rotations, and still small strains,^[^ ^29^ ^]^ or nonlinear numerical schemes such as finite element methods could be an appropriate alternative, especially for the investigation of more complex actuator geometries.

## 3 Conclusion

In summary, the systematic humidity dependent rheological characterization in combination with the measurement of volumetric strains of the active layers allowed to predict the actuator movement of bilayers as simplest geometry. The distinct mechanical properties of the investigated materials composing the properties of the bilayer actuator are rate/time independent at the observed times scales of the actuator movement, which is a few seconds. Thus, neither the diffusive kinetics of the water uptake process nor the viscous part of the materials contribute significantly to the observed actuator movements. The actuator movement is dominated by the volumetric strain and the change in Young's modulus of PEDOT:PSS, both being a function of relative humidity.

Concluding, this contribution goes beyond a phenomenological description and gives physical insights into autonomous actuation behavior. The humidity dependent mechanical behavior presented here can ultimately be incorporated into numerical finite element schemes, which will allow the prediction of arbitrarily shaped geometries and might enable the simulation of the complex movements of plants. Speaking about hierarchical structures and multifunctional behavior of plants, the autonomous switching by humidity, which was attributed in this manuscript to the PSS part of PEDOT:PSS, can be also coupled to active electrical switching enabled by the electronically conducting PEDOT in future studies.

## 4 Experimental Section

### Materials

PEDOT:PSS aqueous dispersion (Clevios PH1000 by Heraeus), NaPSS (Sigma Aldrich, ${\bar{M}}_{w}$ = 70 000 g mol^–1^), PS (Sigma Aldrich, ${\bar{M}}_{w}$ =  192 000 g mol^–1^), ethylene glycol (abcr), and 4‐dodecylbenzenesulfonic acid (Sigma Aldrich) were used as received. The PDMS prepolymer mixture was prepared by mixing base and curing agent (Sylgard 184 by Dow Corning) in a 10:1 weight ratio, followed by vigorous stirring for 2 min. The PEDOT:PSS mixture was prepared by the addition of ethylene glycol (5 vol%) and 4‐dodecylbenzenesulfonic acid (1 vol% of a 0.2 vol% solution in water, i.e., 0.002 vol% of DBSA) to the commercial dispersion as well as sonication (15 min) and filtration (0.2 µm PA filters).

### Typical Manufacture of a Freestanding Bilayer

PEDOT:PSS/PDMS bilayers were made by sequential deposition on a sacrificial NaPSS layer spin coated from a watery solution (4 wt%, 2500 rpm for 30 s) on a precleaned glass slide. PDMS was spin coated onto NaPSS, cured in an oven at 70 °C for 2 h, and then plasma treated (100 W, 3 min, 5 sccm O_2_) to increase hydrophilicity as well as adhesion of the following layer. The PEDOT:PSS mixture was spread on the PDMS layer (25 µL per cm^2^ PDMS surface) by blade coating (0.5 mm s^–1^, 300–400 µm gap, Coatmaster 510 by Erichsen), followed by drying on a hot plate at a setting of 140 °C for 30–40 min. PEDOT:PSS/PDMS bilayers were detached by placing the multilayer stack at an angle in a container and slowly filling up with water. Thereby the NaPSS layer was dissolved and the PEDOT:PSS/PDMS bilayer floated onto the water surface. The free‐standing bilayers were scooped up on a plastic foil and dried in an oven (70°C, 2 h).

To make PEDOT:PSS single layers, PEDOT:PSS was deposited on PDMS and treated as in the manufacture of the bilayers. Floating the bilayer on the surface of deionized water with the PEDOT:PSS surface down caused the active material to take up liquid water and thus expand and delaminate from PDMS. The PDMS layer was carefully removed with tweezers and the PEDOT:PSS layer scooped up using a PTFE watch glass. It was flattened and dried in an oven at 70 °C for 2 h.

### Methods

Actuator testing, QCM, and rheology were performed in measurement chambers allowing to control the relative humidity of the environment (Figure S1, Supporting Information). Actuator movements were followed with a 24.2 Mpx mirrorless digital camera (DSLM, Sony α6500) equipped with a 90 mm macro‐optic (SEL90M28G) to obtain close to frame filling images in intervals of 4–6 s.

### QCM

The water uptake of PEDOT:PSS was determined with a quartz crystal microbalance system. PEDOT:PSS (including 5 vol% ethylene glycol and 0.002 vol% DBSA) was spin coated (3000 rpm for 3 min, then 4000 rpm for 1 min) on gold‐plated quartz crystals (by Inficon, 6 MHz fundamental frequency) precleaned by sonication in acetone (2 × 5 min). After deposition, the crystals were dried on a hot plate (100 °C, 30 min). The frequency shift of the crystal mounted in a front load single sensor (SL‐A0E00 by Inficon) was controlled and monitored by a rate/thickness monitor (STM‐2 by Inficon) at various humidities.

### Rheology

Thermomechanical properties were measured of rectangular samples, which featured a length of ≈30 mm, width of 7–10 mm, and thickness as indicated in the text. A modular, stress‐controlled rheometer (MCR 502 WESP by Anton Paar) with an integrated environmental chamber (CTD 180 by Anton Paar) was used. The temperature in the chamber was controlled by Peltier elements and the humidity by a humidity generator (MHG 100 by ProUmid). The samples were tested by extensional rheology using torque controlled measurements with a universal extensional fixture system (UXF12 by Anton Paar) or force controlled measurements with a solid rectangular fixture system (SRF5 by Anton Paar). Details of the individual measurements are given in the Supporting Information.

## Conflict of Interest

The authors declare no conflict of interest.

## Supporting information

Supporting Information

## Data Availability

Research data are not shared.
