# Supplementary material for: From Understanding Mechanical Behavior to Curvature Prediction of Humidity‐Triggered Bilayer Actuators
Source: Adv Mater. 2021 Jan 20;33(9):2007982. doi: 10.1002/adma.202007982 (PMC11469309; doi:10.1002/adma.202007982)
Supplement: Supplementary file 1 — Supporting Information [file ADMA-33-2007982-s001.pdf]

© 2021 Wiley-VCH GmbH

ADVANCED MATERIALS

Supporting Information

for *Adv. Mater.*, DOI: 10.1002/adma.202007982

From Understanding Mechanical Behavior to Curvature Prediction of Humidity-Triggered Bilayer Actuators

Carsten Dingler, Henry Müller, Matthias Wieland, Dominik Fauser, Holger Steeb, and Sabine Ludwigs**

Supporting Information

From Understanding Mechanical Behavior to Curvature Prediction of Humidity-Triggered Bilayer Actuators

Carsten Dingler, Henry Müller, Matthias Wieland, Dominik Fauser, Holger Steeb, Sabine Ludwigs**

1. Sample Preparation

Manufacture of bilayers:
Free-standing PEDOT:PSS/PDMS bilayers were made by sequential deposition and subsequent detachment from the substrate. PDMS was first coated either directly on glass, on a sacrificial NaPSS layer, or a sacrificial PS layer, followed by the deposition of PEDOT:PSS. All samples were prepared on glass substrates (cut Thermo Scientific Menzel slides) pre-cleaned by sonicating in *i*-propanol, deionized water, and acetone (10 min each), followed by an oxygen plasma treatment (100 W, 10 min, 5 sccm O_2) if a sacrificial layer was used.

The sacrificial NaPSS layer was spin coated from a watery solution (4 wt%, 2500 rpm for 30 s) on a pre-cleaned glass slide. The sacrificial PS layer was spin coated from a solution in toluene (1.3 wt%) at 2500 rpm for 30 s. PDMS was spin coated onto a sacrificial layer or directly on glass, cured in an oven at 70 °C for 2 h, and then plasma treated (100 W, 3 min, 5 sccm O_2) to increase hydrophilicity as well as adhesion of the following layer.

The PEDOT:PSS mixture was spread on the PDMS layer (25 µl per cm^2 PDMS surface) by blade coating (0.5 mm s^{-1}, 300–400 µm gap, Coatmaster 510 by Erichsen), followed by drying on a hot plate at a setting of 140 °C for 30–40 minutes. The actual temperature of the film surface during drying of the deposited PEDOT:PSS was lower (~120 °C) as measured with an IR thermometer.

PEDOT:PSS/PDMS bilayers were detached by placing the multilayer stack at an angle in a container and slowly filling up with water. Thereby the NaPSS layer was dissolved and the PEDOT:PSS/PDMS bilayer floated onto the water surface. The free-standing bilayers were scooped up on a plastic foil and dried in an oven at 70 °C for 2 hours. When PS or no sacrificial layer was used, the multilayer was peeled off from the substrate with a razor blade. Where it was used, PS was removed by immersing the sample into toluene (1 min), followed by drying at 70 °C for 15–20 min.

Film thicknesses were measured by profilometry (Dektak 150 Surface Profiler by Veeco). The film thicknesses of the cured PDMS layers were (11.4 ± 1.1) µm (2750 rpm for 5 min), (25.2 ± 1.0) µm (2825 rpm for 1 min), and (48.3 ± 7.6) µm (1650 rpm for 1 min). The thickness of the PEDOT:PSS layer was (5.5 ± 0.6) µm.

PDMS single layers:
PDMS single layers were prepared on NaPSS sacrificial layers by spin coating the PDMS prepolymer mixture at 1500 rpm, 1000 rpm, or 500 rpm for 1 minute to give film thicknesses of (65.8 ± 5.2), (106 ± 14), and (221 ± 35) µm, respectively. The PDMS samples were detached as before and later measured as prepared, as well as 65 µm films also after subjection to an O_2 plasma and 140 °C as in the manufacture of bilayers.

2. Humidity-Dependent Measurements

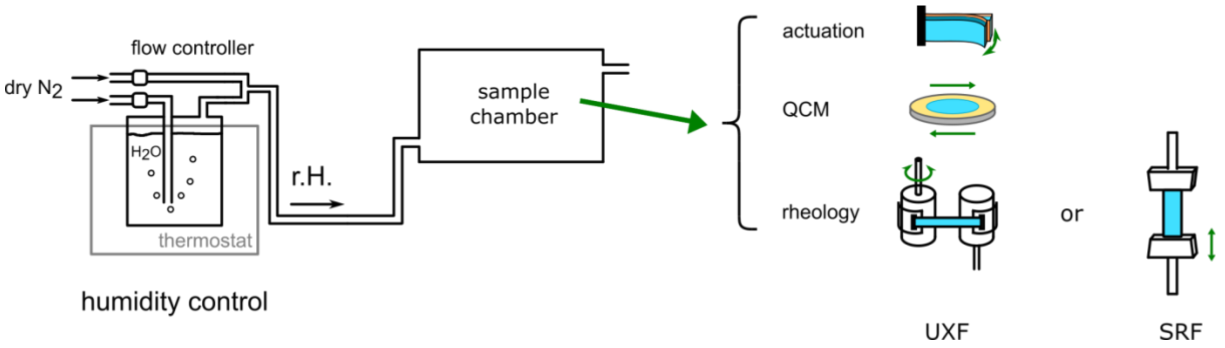

Figure S 1: Sketch of the setup for creating defined relative humidities (r.H.) by passing a stream of dry nitrogen through a wash bottle filled with water and mixing it in various ratios with dry N_2. The stream reaches into chambers where different measurements were conducted, including: i) Actuation: the bilayers were clamped on one side and placed horizontally to be imaged with a digital camera from above. ii) Quartz crystal microbalance (QCM): The change in frequency of an oscillating, circular QCM crystal covered with a thin PEDOT:PSS layer was measured with varying humidity. iii) Rheology: The mechanical properties of samples clamped on two sides were measured in extension by either rotating one of two cylindrical holders (UXF system) or by a vertical movement of the lower of two clamps (SRF system).

Actuators:
To analyze actuation, the bilayers were fixed by a clamp in a glass cell with an inlet and outlet. The samples were placed in a horizontal position (as if standing on their longest edge) to reduce the influence of gravity. The humidity was controlled by a custom-built setup where a stream of dry N_2 was mixed with an N_2 stream saturated with water vapor by passing it through a wash bottle. The temperature of the water in the wash bottle was controlled by a thermostat. The gas flow of the two streams was adjusted by flow controllers (mass-flo® controllers with a PR4000 module by MKS Instruments). The total gas flow was set to 50 sccm in all experiments. The temperature of the thermostat and the volume flow of the water vapor saturated stream were adjusted to reach the desired relative humidities at room temperature, measured with a humidity sensor (HC2A-S by Rotronic). Actuator movements were followed with a 24.2 Mpx mirrorless digital camera (DSLM, Sony α6500) equipped with a 90 mm macro lens (SEL90M28G) to obtain close to frame filling images in intervals of 4–6 s. The images were analyzed by fitting a circle to the actuators with a code written in

Python 3.7.6. The pictures were converted to gray scale in a certain area selected for fitting, where gray-scale values were discarded that were greater than a threshold value adjusted to every image. In this way, bright spots such as the background, in particular, were removed to extract the darker actuators. A circle was then fitted to the actuator using least squares optimization, minimizing the sum of squares of the residuals determined in every iteration step. The residuals consisted of the difference between the distance of a data point to the estimated center and the mean value of all distances. This procedure provided the center of the circle, which was used to calculate the radius *R* as the mean value of distances from the center to all data points. The curvature κ was then obtained as the reciprocal value $\kappa = 1/R$. In order to obtain a smooth transition between fittings of all images of a measurement series, the vertical position of the center of the circle was fixed, and an additional fixed point was added at the contact between bilayer and clamp where appropriate.

QCM:
The sample chamber was placed in an oven (25 or 30 °C) and the humidity controlled by a setup similar to that of the actuator testing. After drying with dry N_2 overnight (also including temporary heating to about 60 °C) the humidity was increased stepwise and the frequency measured. The water uptake

$$w = \frac{m - m_0}{m_0} = \frac{f_0 - f}{f_{\mathrm{blank}} - f_0} \tag{2}$$

as defined by the mass of the water swollen film m and that of the dry film m_0 was calculated according to the Sauerbrey equation[1] using the measured frequency f, the frequency f_0 measured with the dried film, and the frequency f_{blank} of the blank quartz crystal measured before the deposition of PEDOT:PSS. The mixing ratio of the two N_2 streams and the temperature of the water bath were adjusted to reach the desired relative humidities measured with a humidity sensor (HC2A-S by Rotronic).

Rheology:
Two different systems were used to fix and load the samples (cf. Figure S 1). On the one hand, a universal extensional fixture system (UXF12 by Anton Paar) was used and a solid rectangular fixture system (SRF5 by Anton Paar) on the other hand. A static torque (UXF) or force (SRF) was always used to ensure stress in the samples throughout the experiments. A static torque of 100 µNm was generally used with the UXF system and a static force of 20 mN with the SRF holders if not indicated otherwise. Amplitude sweeps were performed at 1 Hz prior to all other measurements to determine the linear viscoelastic region of the samples. A change in humidity did not significantly influence the linear range. Subsequent measurements were conducted using the amplitudes selected beforehand (UXF: bilayer 10 µNm, PEDOT:PSS 10 µNm, PDMS 2 µNm; SRF: bilayer 17 mN). Frequency sweeps were measured from 0.1 to 10 or 100 Hz. However, at $f > 10$ Hz the measurements were dominated by effects caused by the inertia of the measurement tool and sample, so results are exclusively shown from 0.1 to 10 Hz. Humidity sweep experiments with the bilayers were performed with the UXF system at a fixed frequency of 1 Hz. The relative humidity was increased stepwise and multiple data points were measured (and then averaged) during a period of 90 s at each humidity. Amplitude and frequency sweeps were also measured of PEDOT:PSS and PDMS single layers with the UXF system.

Storage modulus *E'* and loss modulus *E''* were generally determined from oscillatory measurements (frequency or humidity sweeps). (Elastic) Young's moduli of bilayers E_{bilayer} were extracted from a linear fit of the stress-strain curve of measurements where an initial force ramp was used. Volumetric strain ε_{vol} and Young's modulus of PEDOT:PSS $E_{\text{PEDOT:PSS}}$ were determined by measurement series with PEDOT:PSS single layers at different relative humidities with the SRF system. Every (quasi-static) humidity step consisted of holding 1 mN to determine the volumetric strain, followed by 5 upwards and 5 downwards force ramps (1–10, 10-15, 15–20, 20–25, 25–30 mN; 0.5 mN s^{-1}) with holding the force constant for 10 min after every ramp. At a given humidity, Young's modulus was calculated in equilibrium for every step from the ratio of stress and strain at the end of the 10 min holding time. To avoid errors from a poor signal-to-noise ratio or non-linear and creep effects, only the values of ramps 2–4 were averaged. The volumetric strain was assumed to be the strain at the minimum force of the initial force ramp at every r.H. The length of the sample at r.H. = 1% was used as initial length l_0. Similar values for ε_{vol} and $E_{\text{PEDOT:PSS}}$ could be obtained with a single upwards force ramp (0.7 mN s^{-1}) followed by holding the maximum force (14 mN) for 30 s at every r.H. During equilibration between all r.H. steps in all measurements, the force was set to zero.
The temperature during all isothermal measurements was 30 °C. After setting another humidity, samples were always equilibrated for 0.5–2 h. Measurements were always performed on at least three different samples and then averaged. Error bars represent the corresponding standard deviation.

3. Actuators

54 µm Actuator

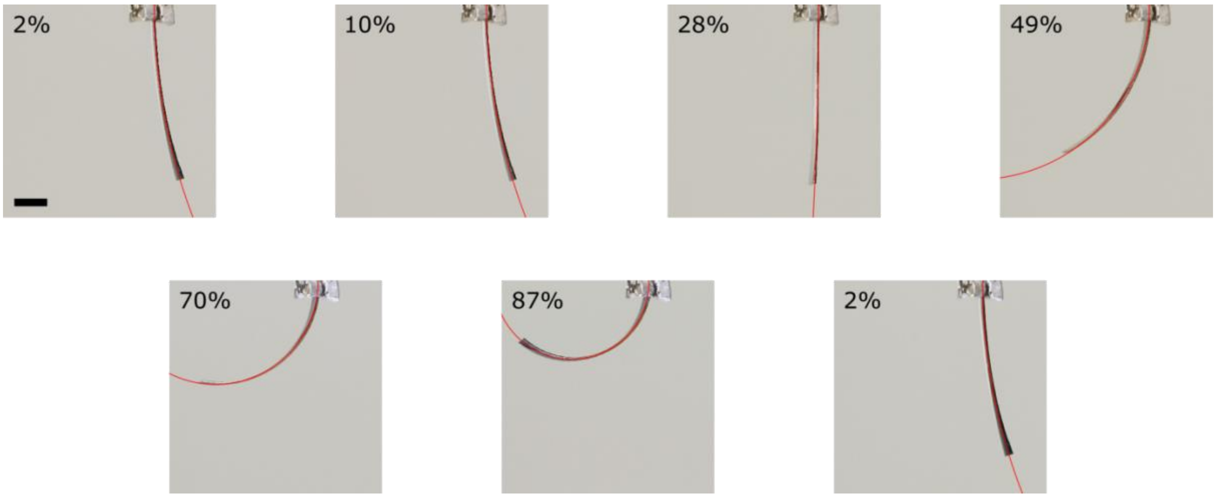

Figure S 2: Fits of the images at various relative humidities of the 54 µm actuator (5.0 mm × 0.7 mm) shown in Figure 2a (scale bar 1mm).

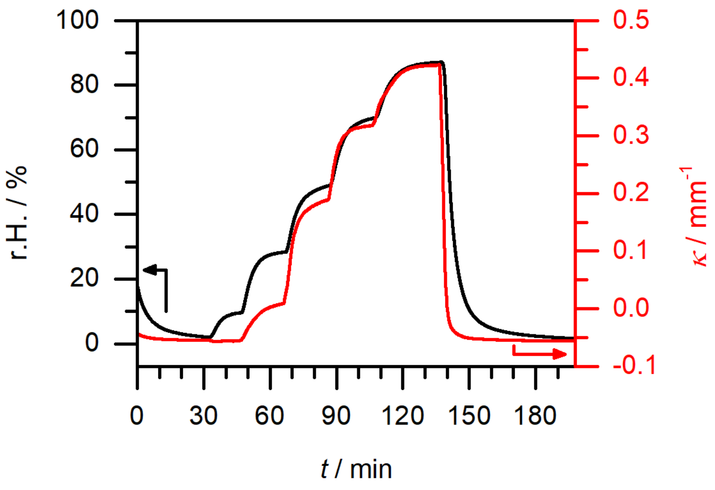

Figure S 3: Course of relative humidity r.H. and curvature κ vs. time t for the actuator measurement in Figure 2a (thickness 54 µm).

17 µm Actuator

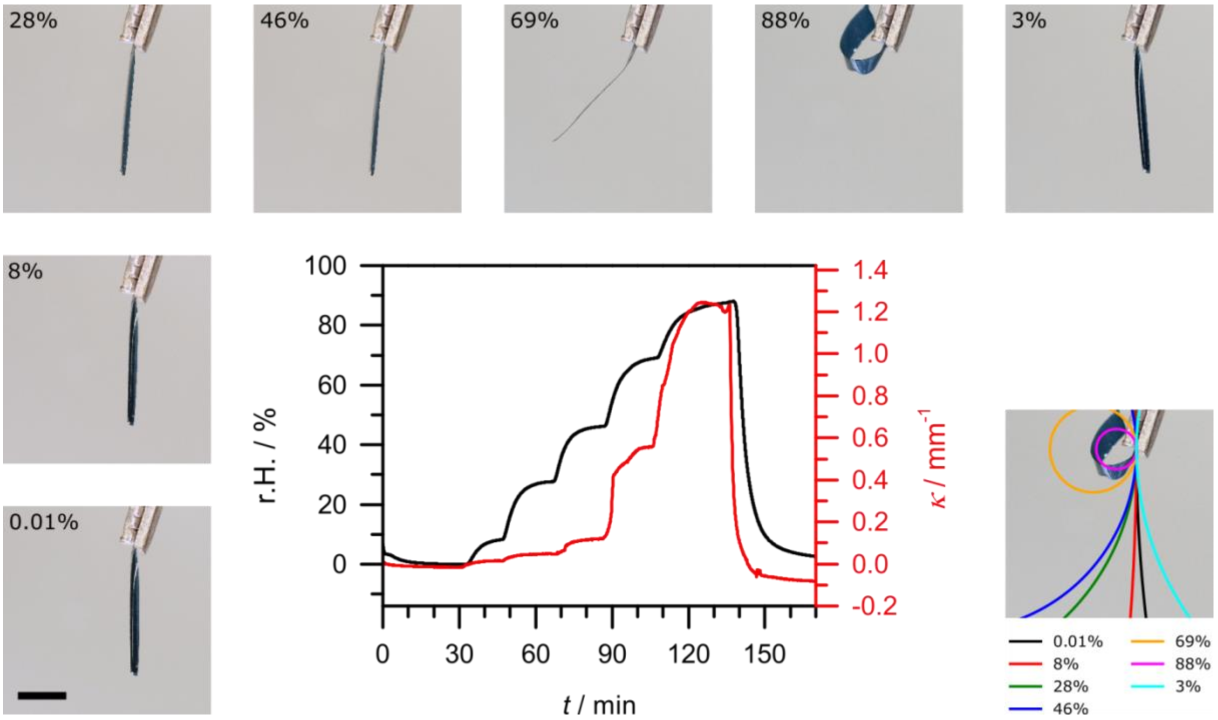

Figure S 4: Actuation of a 17 µm bilayer actuator (5.5 mm × 1.0 mm). The plot of r.H. and curvature κ vs. time t is surrounded by the images taken at the end of the plateaus (scale bar 2 mm). The image on the bottom right shows all fits superposed on the actuator image at r.H. = 88%. The free end of the bilayer had to be excluded from the fits up to r.H. = 69% as it appeared rather straight before it snapped to form a full cycle.

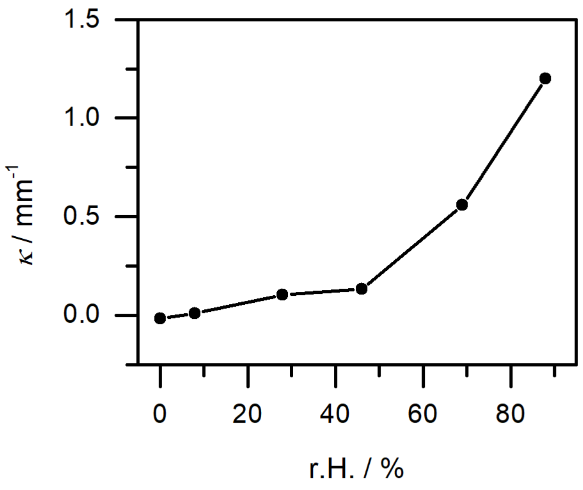

Figure S 5: Curvature κ as a function of relative humidity r.H. extracted from the fits of the 17 µm bilayer actuator shown in Figure S 4.

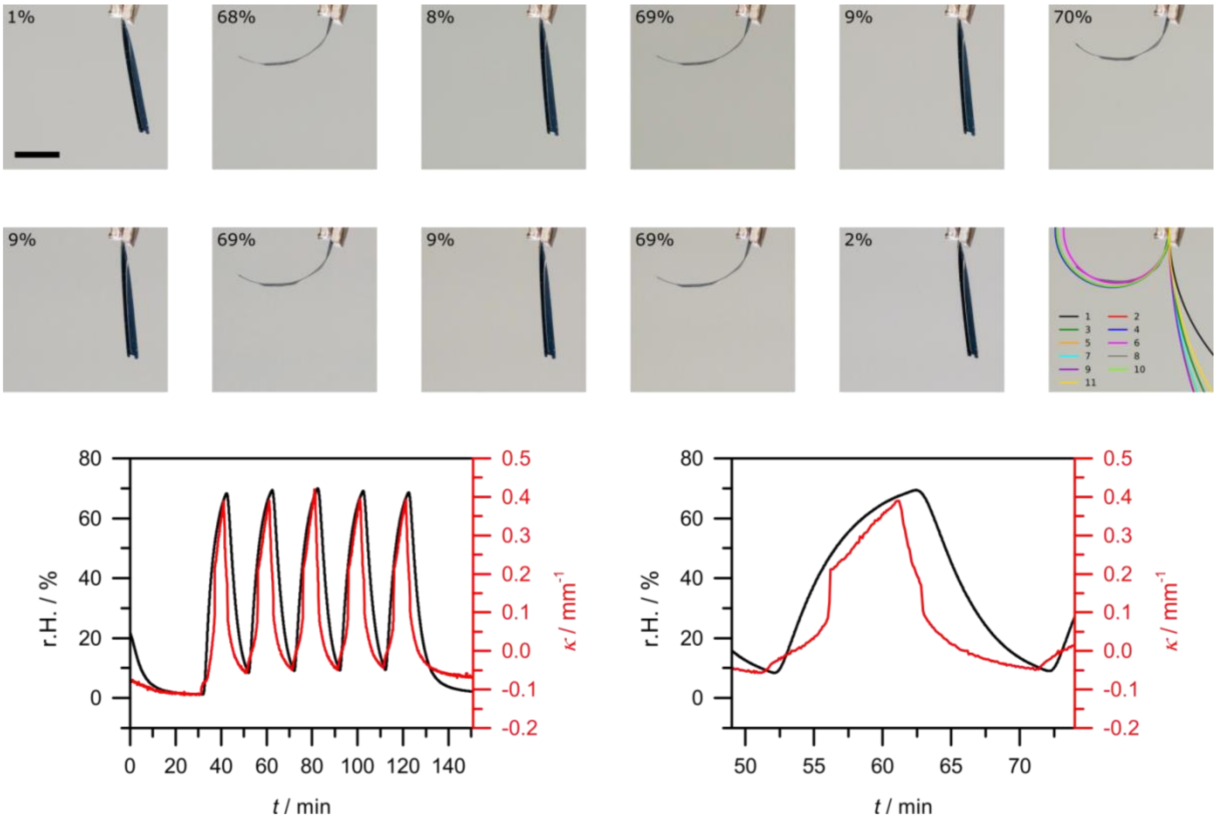

Figure S 6: Actuation of the 17 µm bilayer (5.5 mm × 1.0 mm) when the relative humidity was cycled between about r.H. = 10 and 70%. Top half: Images taken at minimum and maximum curvatures are followed by a compilation of all fits (superposed on the image at r.H. = 70% of the third cycle) with the numbers in the legend indicating the order (scale bar 2 mm). The bottom left image shows the corresponding plot of r.H. and curvature κ vs. time t. The bottom right graph displays a magnification of the second cycle, which shows jumps in κ, likely because of a sudden release of pre-stress.

4. Water Uptake

Table S 1: Water uptake (relative mass increase) of PEDOT:PSS with 5 vol% EG at various relative humidities (r.H.) as measured by QCM at 25 and 30 °C.

r.H. / %	rel. mass increase ($\Delta m/m_0$) / wt%	
	25 °C	30 °C
10	5.2 ± 0.3	6.9 ± 0.2
30	12.3 ± 0.3	13.4 ± 0.2
50	19.6 ± 0.8	19.9 ± 0.7
70	29.9 ± 1.7	29.0 ± 0.4
90	56.6 ± 3.7	51.9 ± 1.2

The number of water molecules per free sulfonate group can be estimated through the parameter $\lambda = n(H_2O)/n(SO_3)$ as calculated in literature.[2] The water uptake with respect to PEDOT:PSS (as in Table S 1) can be related to PSS only by dividing by the weight fraction of PSS in PEDOT:PSS (73 wt%). Including the molar masses of water and of the styrenesulfonate repeat unit gives the number of water molecules per total number of sulfonate groups. However, it is known that the oxidation level of PEDOT is usually about 1/3,[3] which implies that about 18% of the sulfonate groups is blocked by strong ionic interactions with PEDOT. Dividing the number of water molecules per total number of sulfonate groups by 82% therefore gives the number of water molecules λ per free sulfonate group.

5. Rheology with the UXF System

The UXF system consisted of two drums, one of which was attached to the upper rotating shaft of the rheometer and the other one to the stationary rheometer stand. The samples were fixed by the clamps of the drums. By rotating one of the drums, torque-controlled extensional rheology of the polymer films could be conducted. To this end, an oscillating torque was applied to the upper shaft, superposed on a static torque (which relates to uniaxial strain in the sample) to ensure stress in the samples throughout the experiments. All experiments in Figure 2 in the main text were measured with the UXF system.

5.1 Torque-Controlled Amplitude Sweeps

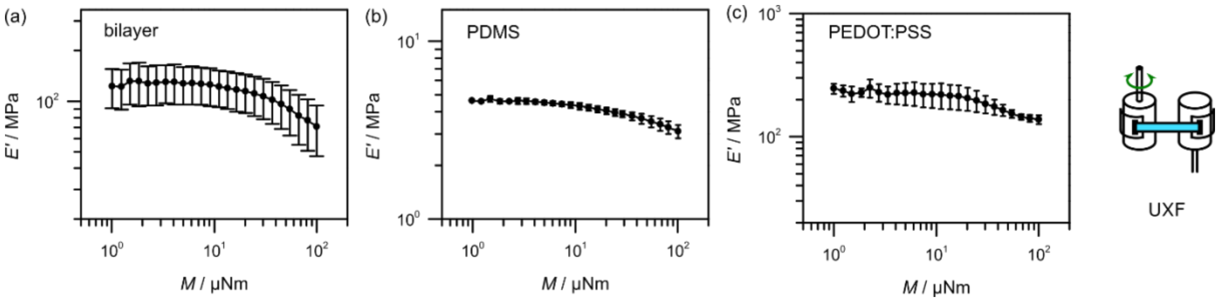

Figure S 7: Averaged amplitude sweeps of (a) 17 µm bilayers, (b) PDMS single layers with a thickness of 65 µm (after plasma treatment and 140 °C), and (c) PEDOT:PSS single layers. All measurements were performed with the UXF system at 30 °C, r.H. = 30%, and with a frequency of 1 Hz.

5.2 Frequency Sweep of PEDOT:PSS

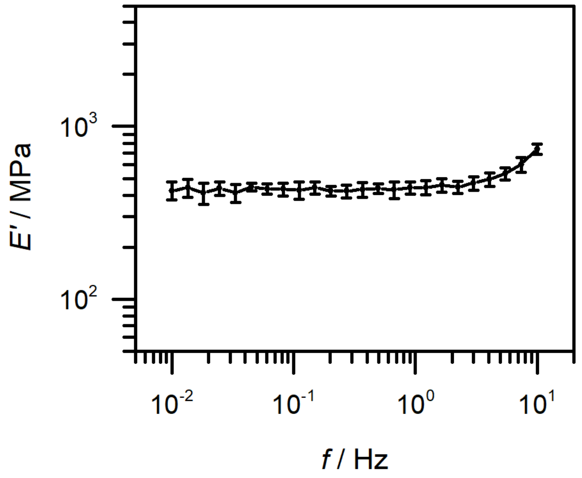

Figure S 8: Averaged frequency sweeps of PEDOT:PSS single layers at r.H. = 30% down to 0.01 Hz.

5.3 Loss in the Materials

The loss modulus of the bilayers seemed to be rather unaffected by a change in humidity (Figure S 9). Adopting a value of 5–6 MPa, *E''* is 1–2 orders of magnitude lower than *E'*. This expectedly confirms that the elastic part (described by *E'*) dominates over the viscous part (described by *E''*) in the viscoelastic PEDOT:PSS/PDMS bilayer. As *E'* diminishes with increasing humidity while *E''* is almost constant, the loss factor of this material—defined as $\tan(\delta) = E''/E'$—increases with increasing relative humidity. At r.H. = 10%, $\tan(\delta)$ adopts a value of $4.4 \cdot 10^{-2}$ but rises to $1.1 \cdot 10^{-1}$ when the relative humidity is increased to r.H. = 90%. This implies that the ratio of viscous to elastic behavior changes as the PEDOT:PSS layer takes up water.

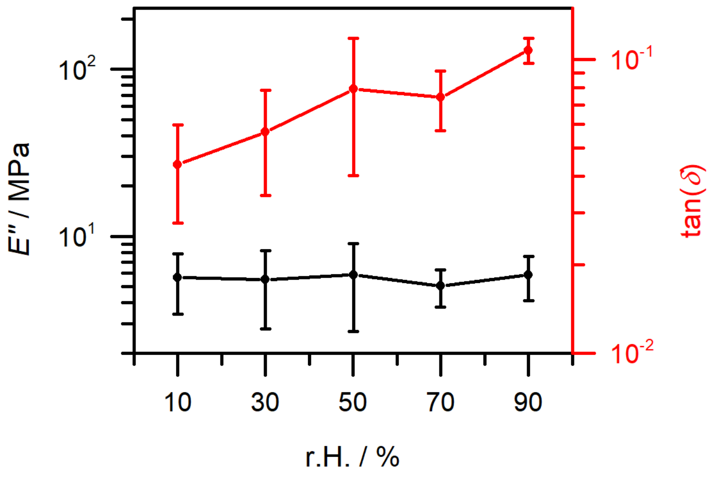

Figure S 9: Loss modulus *E''* and loss factor $\tan(\delta)$ as a function of relative humidity r.H., corresponding to Figure 2e.

In all measurements—also of PEDOT:PSS or PDMS single layers—the loss factor was 10^{-1} or less. This implies that the loss modulus *E''* was always at least one order of magnitude lower than the storage modulus *E'*. Therefore, it approximately holds that $E' \approx |E^*| \approx E$, so Young's modulus could be approximated by the storage modulus.

5.4 Thickness Dependence of the Mechanical Properties of PDMS

Since the determination of the elastic moduli of the 48 µm and the 11 µm PDMS films was experimentally challenging, films with thicknesses between 65 and 220 µm were experimentally characterized. First, PDMS films without any post-treatment were measured, i.e. no plasma and no high-temperature treatment (Figure S 10) was applied.

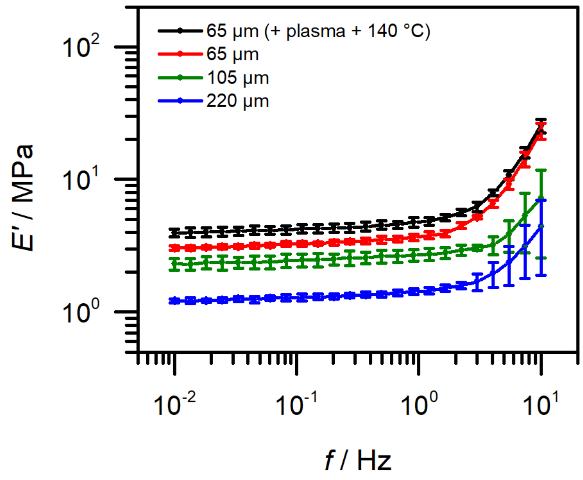

Figure S 10: Averaged frequency sweeps of PDMS single layers with distinct thicknesses at r.H. = 30%, measured down to 0.01 Hz.

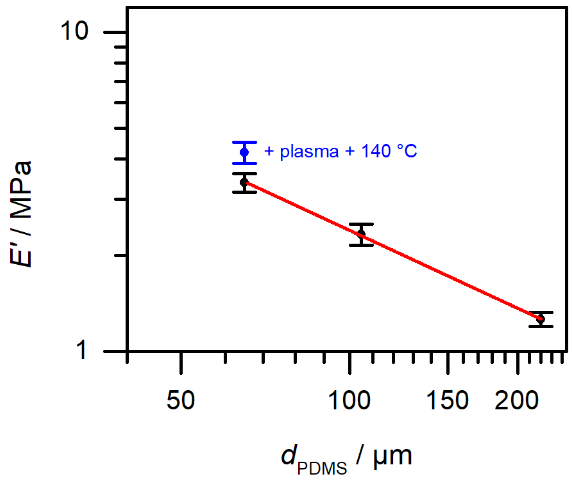

Figure S 11: Storage modulus of thin PDMS films (extracted at 0.1 Hz) as a function of PDMS thickness in a double logarithmic plot. The solid red line indicates a linear fit to the data points without plasma and 140 °C treatment.

It can be observed that all frequency sweep curves of the different films showed a similar trend but were vertically shifted with respect to one another (Figure S 10). Selected storage moduli *E'* at 0.1 Hz are plotted as a function of thickness in Figure S 11. At 0.1 Hz the storage modulus increased from 1.3 to 2.3 and 3.4 MPa for PDMS with a thickness of 220, 105, and 65 µm, respectively.

The dependence on thickness may be caused by dissimilar thermal progression during curing. However, it may also be due to different shear loads[4] as higher spin speeds were used to prepare thinner films by spin coating. From the report of Liu et al.[4] it can be followed that the data could be interpolated linearly in a double-logarithmic plot as indicated in Figure S 11.

Since the 48 µm thick PDMS layers were prepared in an identical way, only slightly extrapolating the trend would give a (bulk) storage modulus of 4.3 MPa. For the preparation of the 11 µm thin films, the spin coating conditions had to be adjusted. The spin time was required to be longer, while spin speeds were chosen smaller. Koschwanez et al.[5] showed that the PDMS thickness depends on the spin speed via a power function, so the theoretical spin speed at the same spin time as used with the thicker samples could be calculated. With this assumption *E'* could be estimated to be about 5.8 MPa for the 11 µm thickness.

Influence of Post-Treatments on the Moduli

Figure S10 also shows data for the 65 µm thick layer after plasma and additional temperature treatment at 140 °C. The elastic modulus *E'* was 4.2 MPa at 0.1 Hz, i.e., slightly higher than without any treatment (3.4 MPa). This could be the consequence of the plasma treatment or the temperature treatment. However, it cannot be excluded that high-temperature and plasma treatment had a synergistic effect on increasing the moduli as well.

Plasma treatment

To estimate the effect of plasma treatment, a PDMS sample could be regarded as a bilayer with a thick bulk and a thin surface oxide layer. According to literature,[6] the surface layer should be in the nm range (< 100 nm), given the plasma dose (time × power) used in this work. As all PDMS layers in this investigation were no less than 11 µm thick and therefore at least two orders of magnitude thicker than the assumed surface layer, the thickness of the bulk is approximately equal to the total thickness.

In a very simplified approach, the effective modulus of this bilayer can be calculated as a volume fraction-weighted sum of the bulk modulus and the surface-layer modulus. If it is assumed that the increase in *E'* of the 65 µm layer in Figure S 11 merely results from plasma (and not high-temperature) treatment, these data can be used to eliminate the thickness and the modulus of the surface oxide layer in the calculation of the effective modulus of any other thickness.

Using the moduli determined above (4.3 MPa for the 48 µm thick film and 5.8 MPa for the 11 µm thick film) as bulk moduli, effective moduli of the post-treated films of 5.5 MPa for the 48 µm thick PDMS and 11 MPa for the 11 µm thick PDMS films could be estimated.

Heat treatment

The normal curing temperature for PDMS was 70 °C. However, an additional heat treatment of 140°C was necessary after deposition of the PEDOT:PSS layer. Actual sample temperatures were ~120 °C as mentioned in the Experimental Part. Johnston et al.[7] found a strong dependence of Young's modulus on the curing temperature, so it is possible that thermal post treatment led to additional curing of PDMS. Based on the authors' suggested linear regression,[7] the modulus would increase by a factor of 1.26 or 1.37 when increasing the curing temperature from 70 to 120 °C or 140 °C, respectively.

Multiplying the bulk modulus of 4.3 MPa of the 48 µm thick film by a factor of 1.26 or 1.37 would give 5.4 or 5.9 MPa, respectively. A similar calculation for the 11 µm thin PDMS would provide values of 7 or 8 MPa, respectively.

In summary, combining the estimations with plasma and thermal post-treatment, Young's modulus E_{PDMS} may be assumed to be around 5 MPa for 48 µm thin PDMS, while 10 MPa is assumed for the 11 µm thickness.

6. Rheology with the SRF System

With the SRF system, a linear drive unit at the bottom of the rheometer was used to stretch the samples linearly, rather than by winding up on a drum. The samples were fixed by clamps of a solid rectangular fixture system (SRF5 by Anton Paar). An oscillating force was applied by the linear motor and also superimposed on a static force to ensure tensile stress in the sample throughout. The static force was approached with a force ramp of 1 mN s^{-1} to avoid abrupt stress in the samples. Measurements in Figure 3 of the main text were taken with the SRF system. A direct (quantitative) comparison of the measurements with the UXF and SRF systems is difficult because the loading forces were different as well as sample dimensions such as the aspect ratio.

6.1 Amplitude Sweeps

Force-controlled amplitude sweeps with the SRF system showed fairly linear behavior in all force ranges tested (Figure S 12). Medium force amplitude and static force were therefore chosen as the signal-to-noise ratio was poor in the lower range and samples ruptured when loaded with higher forces for extended periods of time. It should be noted, however, that a dependence on the amount of static force was observed (Figure S 12).

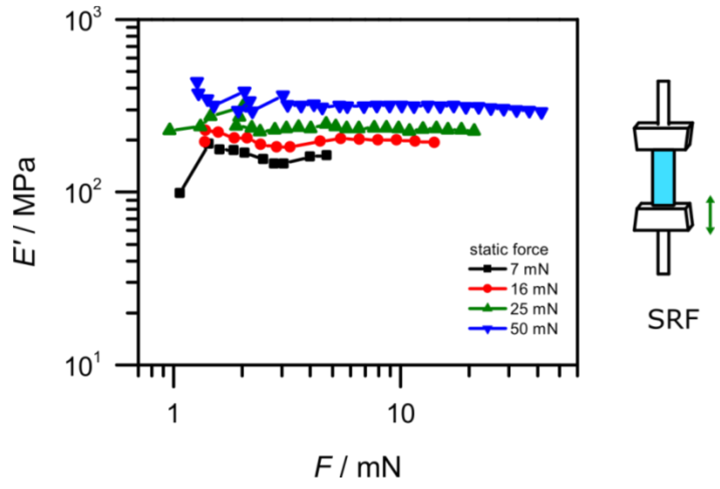

Figure S 12: Averaged amplitude sweeps of 17 µm bilayers with the SRF system at 1 Hz and distinct static forces. Error bars were omitted as they were larger than the variation caused by a different static force.

6.2 Bilayers with Different Thicknesses

A comparison of averaged frequency sweeps of bilayer samples with different PDMS thickness is shown in Figure S 13. The corresponding Young's moduli were also measured here (cf. Figure S 14) and are presented in Table S 2. A clear trend of decreasing storage modulus with increasing bilayer thickness is evident. The main reason for it is the increasing percentage of the softer PDMS layer, which leads to a lower effective modulus. This is supported by PDMS itself becoming softer when it gets thicker as discussed above, leading to an even lower effective modulus.

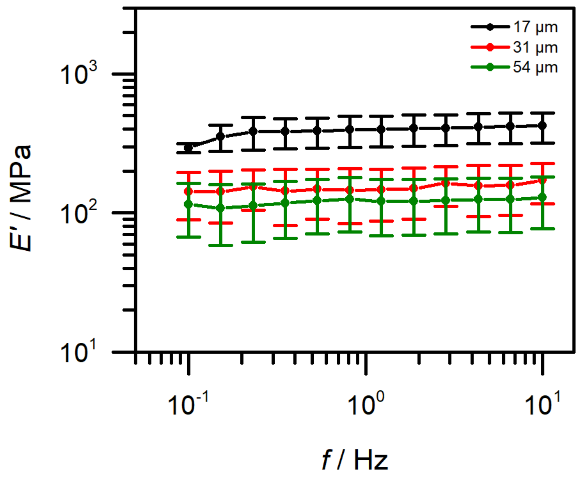

Figure S 13: Comparison of averaged frequency sweeps of bilayers with distinct total thicknesses at r.H. = 30%.

Table S 2: Young's moduli *E* and storage moduli *E'* of 17 µm bilayers at r.H. = 30% extracted from the measurements shown in Figure S 13 and Figure S 15. The principle of these measurement is illustrated in Figure S 14.

Sample	*E* / MPa	*E'* (at 1 Hz) / MPa
without EG (17 µm)	624 ± 173	736 ± 182
17 µm	316 ± 92	398 ± 99
31 µm	107 ± 68	147 ± 60
54 µm	91 ± 45	121 ± 53

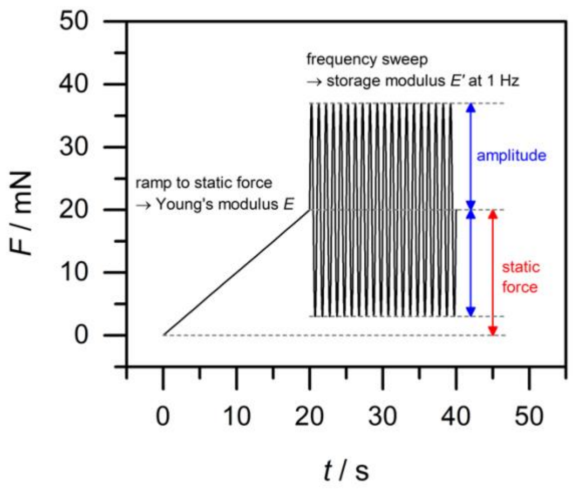

Figure S 14: Schematic depiction of the measurements shown in Figure S 13 and Figure S 15, where Young's modulus could be extracted from the first part of the sequence (ramp) and the storage modulus from the second one (frequency sweep).

6.3 Bilayers without Ethylene Glycol

Bilayers (17 µm) without ethylene glycol added to PEDOT:PSS during processing featured a Young's modulus of E = 624 MPa, which is about double the value with EG (316 MPa). Therefore, the addition of ethylene glycol (probably without complete removal during high-temperature treatment) led to a decrease in storage modulus of the bilayer samples by a factor of about 2 (Table S 2 and Figure S 15).

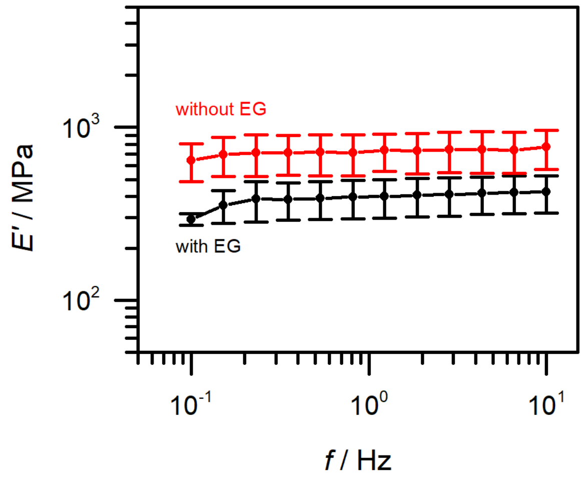

Figure S 15: Comparison of averaged frequency sweeps of 17 µm bilayers prepared with or without ethylene glycol (r.H. = 30%).

6.4 Measurement of Volumetric Strain and Young's Modulus of PEDOT:PSS

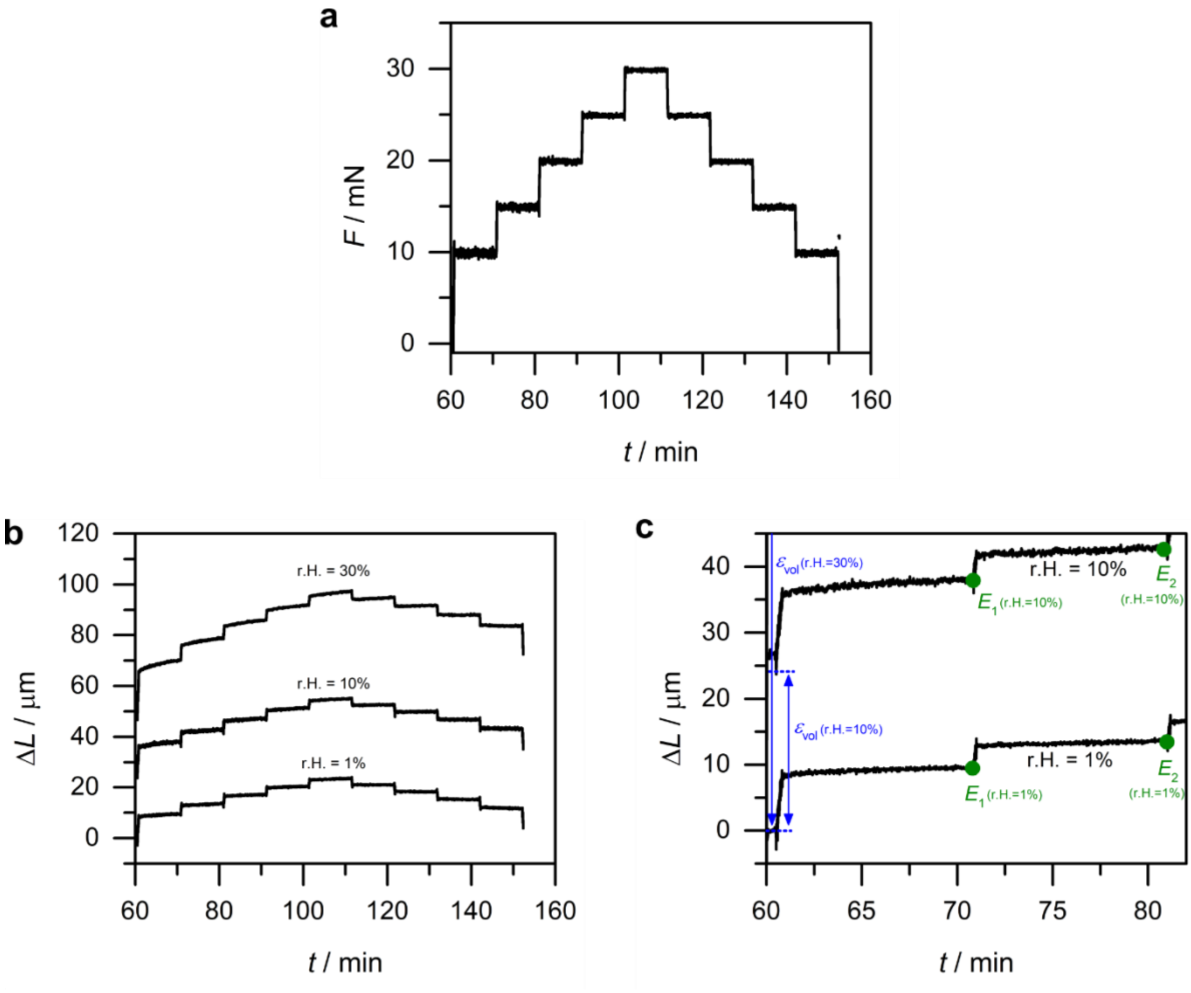

Figure S 16: Measurements to determine ε_{vol} and $E_{PEDOT:PSS}$ for the model with multiple force ramps per r.H. (a) Exemplary force ramps (r.H. = 30%) after initial drying, with holding the force after every ramp. (b) Measured displacement during all steps at three humidities. (c) Displacement of the first two steps at r.H. = 1 and 10% with indication of how to determine ε_{vol} and $E_{PEDOT:PSS}$.

Volumetric strains and the Young's modulus of PEDOT:PSS were determined at distinct humidities by loading PEDOT:PSS single films with force ramps and holding the maximum force of every step for a certain period of time (Figure S 16a). The resulting displacements were measured (Figure S 16b) and ε_{vol} and $E_{PEDOT:PSS}$ calculated as indicated in Figure S 16c. The samples were always equilibrated to a changed r.H. with the force set to zero and the length of the sample determined at 1 mN to ensure the sample is straight. The volumetric strains ε_{vol} were calculated from the sample lengths l at every step and the initial length l_0 at r.H. = 1%. Young's modulus of PEDOT:PSS was determined at every step from force and displacement at the end of the force plateaus (after a possible creep). For a given humidity, the moduli $E_{PEDOT:PSS}$ of steps 2 to 4 were averaged to obtain the best signal to noise ratio (step 1 excluded) and eliminate errors from nonlinear and creep effects (step 5 and other steps

of downward ramps). Similar values of ε_{vol} and $E_{PEDOT:PSS}$ could be obtained with only a single force ramp per r.H. and calculating ε_{vol} from the strain at 1 mN as well as $E_{PEDOT:PSS}$ from a linear fit to the stress-strain curves (Figure S 17).

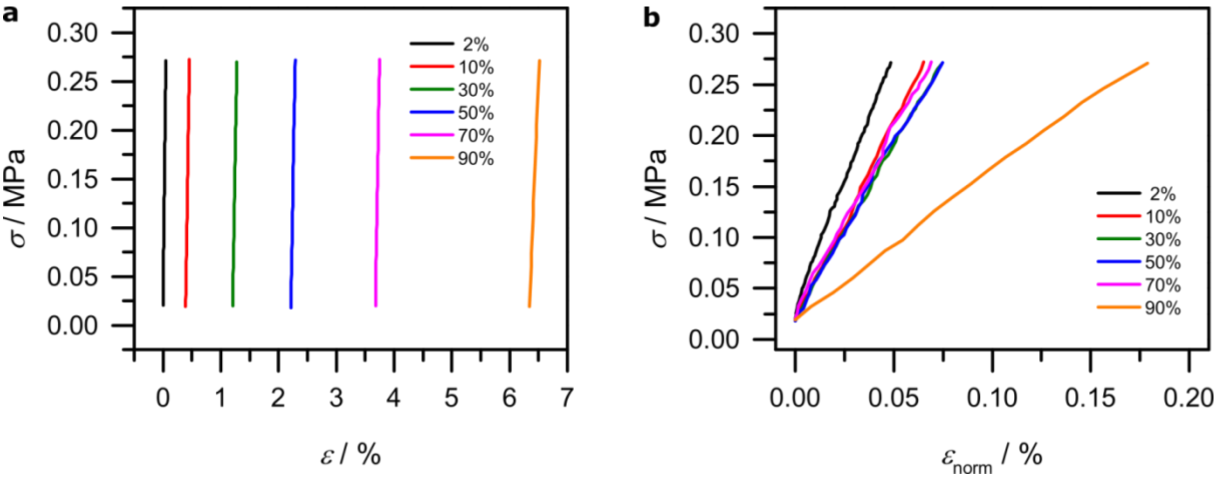

Figure S 17: Measurements to determine ε_{vol} and $E_{PEDOT:PSS}$ for the model with a single ramp per r.H. step. (a) Stress-strain curves for a PEDOT:PSS single layer at various relative humidities. (b) Corresponding curves with normalized strain, which implies that the curves from (a) were shifted so that the initial strains were zero.

7. Modeling

7.1 General Model Description

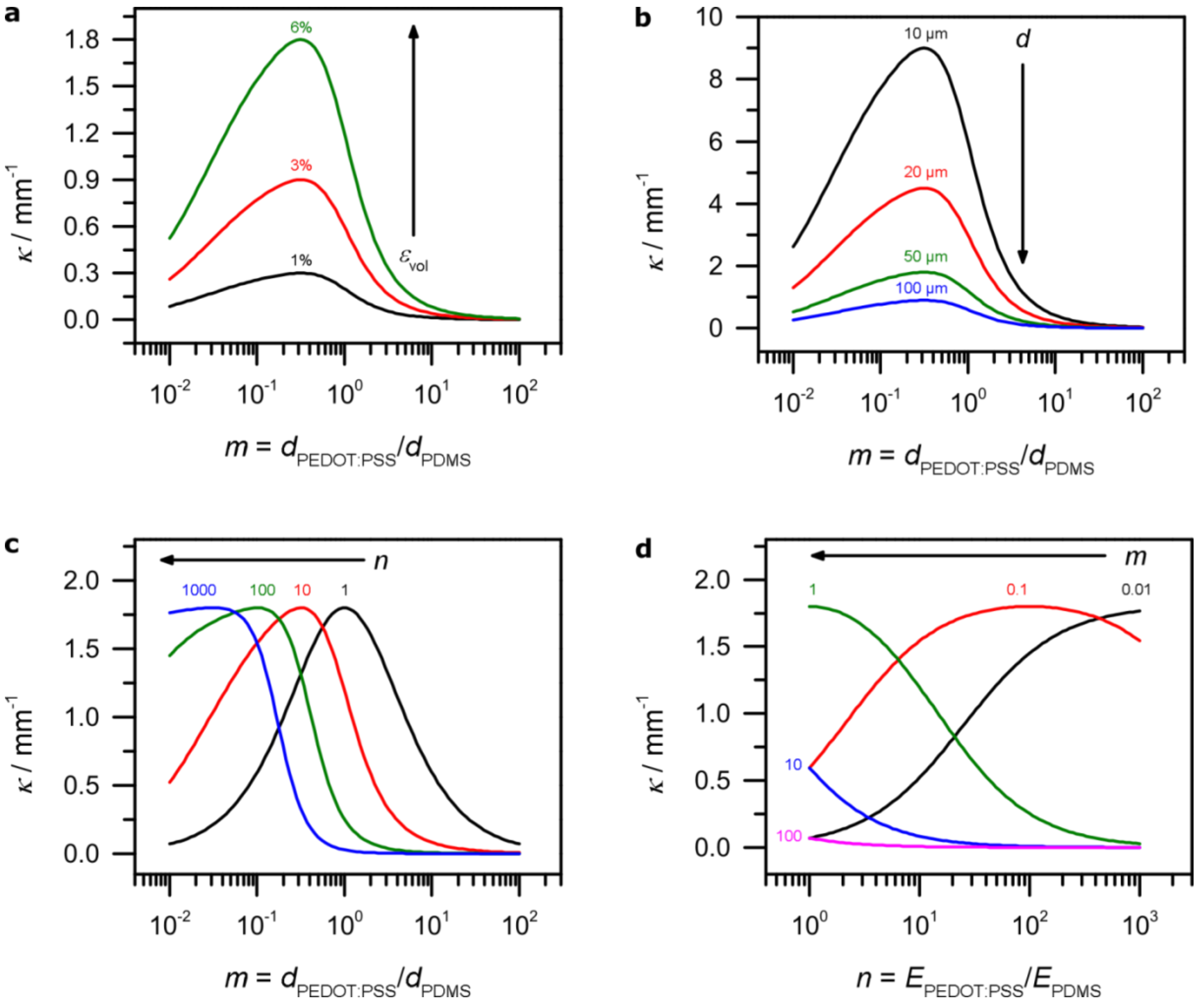

Figure S 18: General 2D profiles of the curvature as a function of distinct parameters according to the Timoshenko equation. The calculations were performed with n = 10, d = 50 µm, and ε_{vol} = 6% where not varied. Curvatures are displayed as a function of the thickness ratio m with varying (a) volumetric strain ε_{vol}, (b) total thickness d, and (c) moduli ratio n, as well as (d) as a function of n with varying m.

7.2 Actuator Design of a 54 µm Bilayer

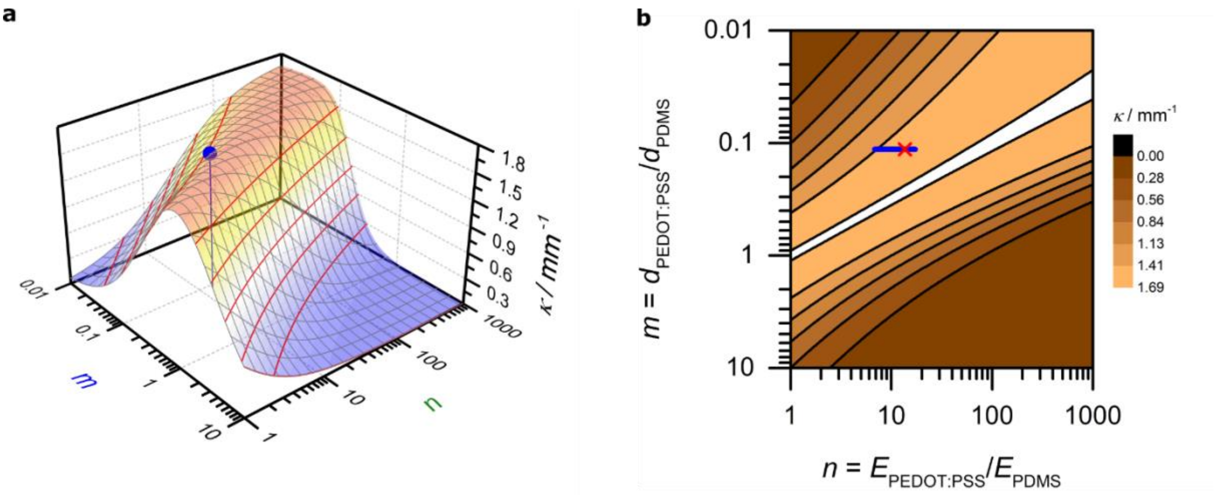

Figure S 19: (a) 3D Plot of the curvature of a 54 µm bilayer predicted by the linear Timoshenko composite beam equation using ε_{vol} = 6.1% and d = 54 µm. The blue dot highlights the data point at the experimental values m = 0.11 and n = 14 (E_{PDMS} = 5 MPa, $E_{PEDOT:PSS}$ = 68 MPa). (b) Corresponding contour plot. The blue line corresponds to the expected range for the experimental actuators with a thickness ratio m = 0.11 and the moduli ratio n = 6.8–17 ($E_{PEDOT:PSS}$ = 68 MPa, the modulus of PDMS E_{PDMS} was chosen to be between 4–10 MPa to show the maximum anticipated range). The red cross highlights the data point with n = 14 on the blue line.

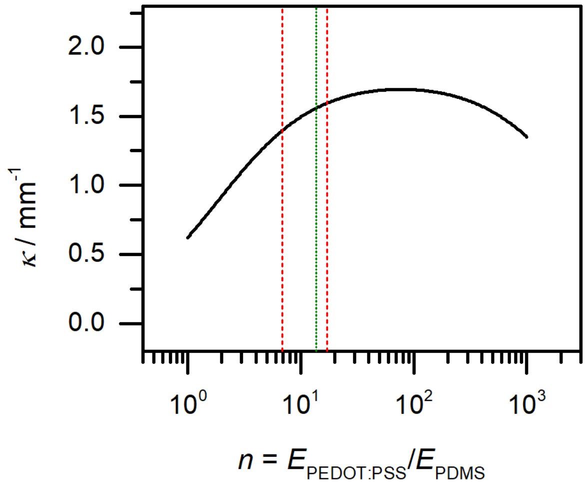

Figure S 20: Curvature as a function of moduli ratio n for a 54 µm bilayer with ε_{vol} = 6.1% and thickness ratio m = 0.11 (corresponds to a cross section of the 3D plot in Figure S 19 at m = 0.11). In addition, the expected limits for the experimental actuators of n = 6.8 and 17 (E_{PDMS} = 10 and 4 MPa, $E_{PEDOT:PSS}$ = 68 MPa) are highlighted by the dashed red lines and the assumed value of n = 14 (E_{PDMS} = 5 MPa) by the dotted green line.

7.3 Actuator Design of a 17 µm Bilayer

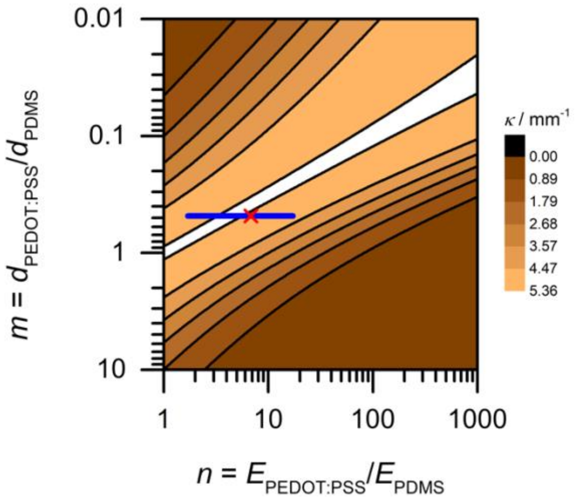

Figure S 21: Contour plot of the curvature κ as a function of thickness ratio m and moduli ratio n for a 17 µm bilayer with ε_{vol} = 6.1%, corresponding to the 3D plot in Figure 3. The expected maximum range of the experimental actuators with m = 0.48 and n = 1.7–17 ($E_{PEDOT:PSS}$ = 68 MPa, the modulus of E_{PDMS} was chosen to be 4–40 MPa to show the maximum anticipated range) is highlighted by a blue line. The red cross highlights the data point with n = 6.8 (E_{PDMS} = 10 MPa, $E_{PEDOT:PSS}$ = 68 MPa) on the blue line.

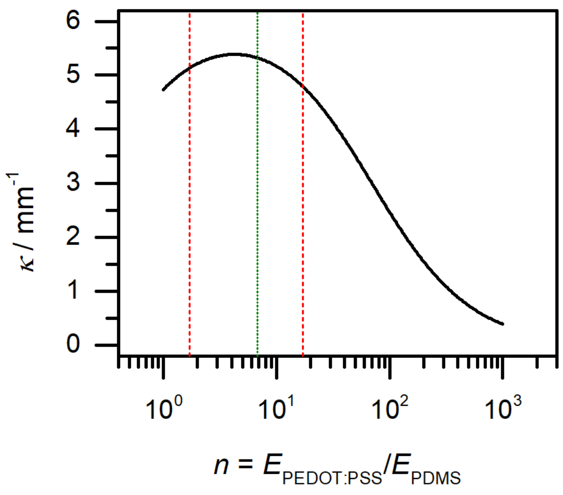

Figure S 22: Curvature κ as a function of moduli ratio n for 17 µm bilayers with ε_{vol} = 6.1% and m = 0.48 (corresponds to a cross section of the 3D plot in Figure 3c at m = 0.48). The expected maximum limits of the experimental bilayers of n = 1.7 and 17 (E_{PDMS} = 40 and 4 MPa, $E_{PEDOT:PSS}$ = 68 MPa) are highlighted by dashed red lines, and the assumed value of n = 6.8 (E_{PDMS} = 10 MPa) by the dotted green line.

7.4 Actuator Visualization

The calculated curvatures were visualized with a code written in Python 3.7.6. First, the number of windings N of an actuator was calculated from the curvature κ and the length L according to

$$N = \frac{L\,\kappa}{2\,\pi}\ . \tag{3}$$

As an actuator cannot penetrate itself if $N > 1$ (i.e., when if forms more than a full circle), it could not exactly be displayed as a circle. Instead, the actuators were visualized as an (Archimedian) spiral according to the equations

$$x = r\cos(\theta) \qquad\qquad y = r\sin(\theta) \tag{4}$$

for the x- and y-coordinates, with the radius r being a linear function of the angle θ

$$r = a\,\theta\ . \tag{5}$$

The constant a represents a factor determining the length of the spiral, which can be calculated according to

$$a = 2\,L\left(\int_{\theta_1}^{\theta_2}\sqrt{1+\theta^2}\,\mathrm{d}\theta\right)^{-1} \tag{6}$$

giving a spiral of the desired length L. The starting and final angle θ_1 and θ_2 determine the beginning and ending of the spiral. As an actuator tends to curl up inward, the final angle θ_2 represents the part of the actuator attached to the clamp. It was therefore fixed to

$$\theta_2 = c\ \cdot 360°\ . \tag{7}$$

The starting angle θ_1 is therefore the one that depends on the "degree of bending" of the actuator and was thus calculated from the number of windings N according to

$$\theta_1 = (c - N)\ \cdot 360°\ . \tag{8}$$

The factor c represents a constant determining how close the individual windings of the spiral should be ($c > N$). However, for a realistic depiction of an actuator, the line thickness would have to be so thin that it is not visible. Therefore, the line thickness was chosen to be thicker, but the spacing of the windings had to be adjusted accordingly. As a consequence, in the visualizations, the actuators show a higher radius (lower curvature) on the outside and a lower radius (higher curvature) on the inside of the spiral than an ideal depiction featuring a line thickness actually corresponding to the actuator thickness would show.

7.5 Maximum Range of Predicted Curvatures

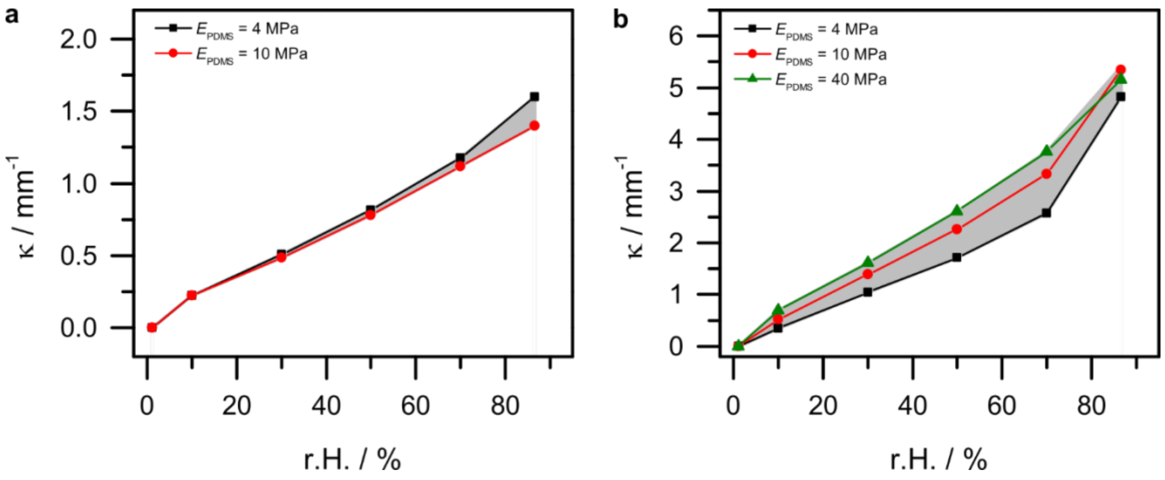

Figure S 23: (a) Curvature κ as a function of r.H. for a 54 µm actuator as predicted by the Timoshenko equation using m = 0.11 and distinct E_{PDMS} with $E_{PEDOT:PSS}$ as well as ε_{vol} being a function of r.H. (as shown in Figure 3b). All possible curvatures in a range from E_{PDMS} = 4 to 10 MPa are indicated by the gray area. (b) Corresponding plot for a 17 µm bilayer (m = 0.48) with the gray area indicating all possible curvatures in the range from E_{PDMS} = 4 to 40 MPa.

References

[1] G. Sauerbrey, *Z. Phys.* **1959**, *155*, 206–222.

[2] M. Wieland, C. Dingler, R. Merkle, J. Maier, S. Ludwigs, *ACS Appl. Mater. Interfaces* **2020**, *12*, 6742–6751.

[3] G. Zotti, S. Zecchin, G. Schiavon, F. Louwet, L. Groenendaal, X. Crispin, W. Osikowicz, W. Salaneck, M. Fahlman, *Macromolecules* **2003**, *36*, 3337–3344.

[4] M. Liu, J. Sun, Y. Sun, C. Bock, Q. Chen, *J. Micromech. Microeng.* **2009**, *19*, 35028.

[5] J. H. Koschwanez, R. H. Carlson, D. R. Meldrum, *PLoS One* **2009**, *4*, e4572.

[6] a) F. A. Bayley, J. L. Liao, P. N. Stavrinou, A. Chiche, J. T. Cabral, *Soft Matter* **2014**, *10*, 1155–1166; b) B. Sarrazin, R. Brossard, P. Guenoun, F. Malloggi, *Soft Matter* **2016**, *12*, 2200–2207; c) S. Béfahy, P. Lipnik, T. Pardoen, C. Nascimento, B. Patris, P. Bertrand, S. Yunus, *Langmuir* **2010**, *26*, 3372–3375.

[7] I. D. Johnston, D. K. McCluskey, C. K. L. Tan, M. C. Tracey, *J. Micromech. Microeng.* **2014**, *24*, 35017.
